# Supplementary figures and images for: Germline stem cell integrity and quiescence are controlled by an AMPK-dependent neuronal trafficking pathway
Source: PLoS Genet. 2023 Apr 14;19(4):e1010716. doi: 10.1371/journal.pgen.1010716 (PMC10132661; doi:10.1371/journal.pgen.1010716)

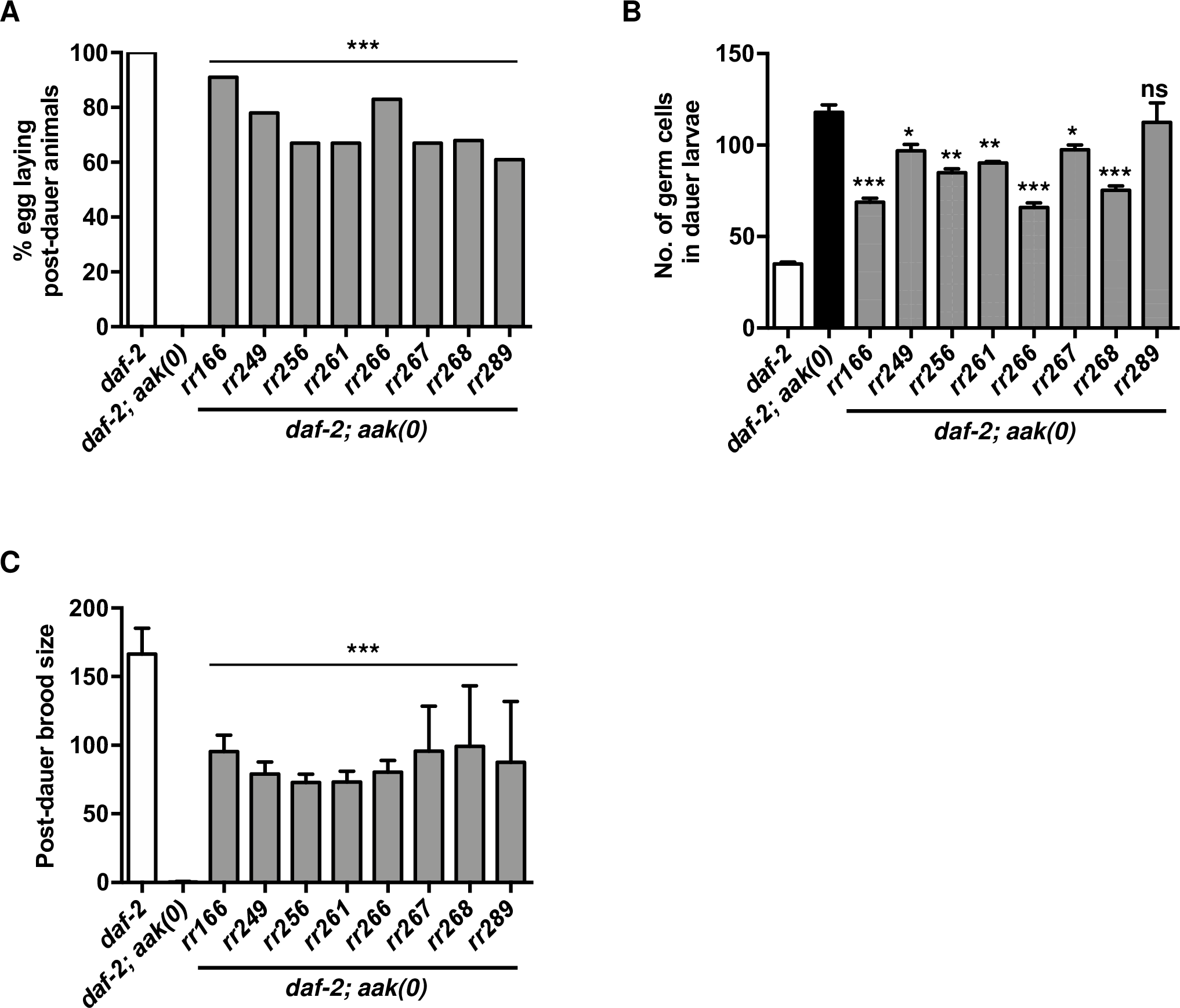

Supplement: S1 Fig — (A-C) Mutants isolated from an EMS suppressor screen partially suppress the (A) post-dauer sterility, (B) dauer germline hyperplasia, and (C) brood size defects associated with a lack of AMPK signalling. ***P < 0.0001, **P < 0.001, *P < 0.05 when compared to daf-2; aak(0) using ordinary one-way ANOVA for post-dauer brood size and no. of germ cells in dauer larvae. ***P < 0.0001 when compared to daf-2; aak(0) using Marascuilo procedure for % egg laying post-dauer animals. All animals isolated from the screen include daf-2; aak(0) in the background. The values for % egg laying post-dauer animals, no. of germ cells in dauer larvae, and post-dauer brood size are presented as means. Each assay was repeated three times with 50 animals in each trial. n = 50. (TIF) [file pgen.1010716.s001.tif]

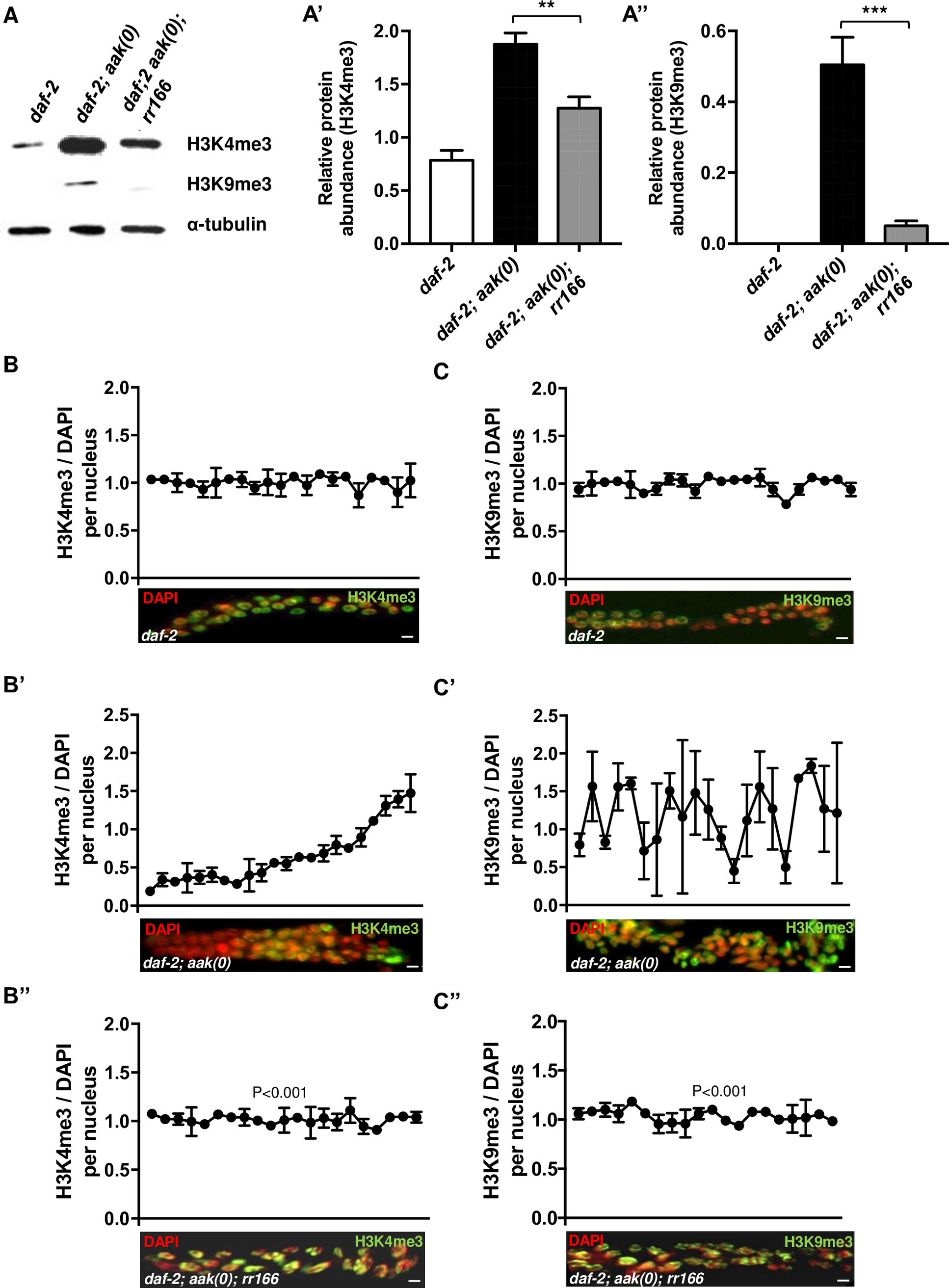

Supplement: S2 Fig — (A) Global levels of H3K4me3 and H3K9me3 were quantified by performing whole-animal western analysis on dauer larvae. (A’-A”) Levels of chromatin marks were quantified and normalized to α-tubulin using ImageJ software. ***P < 0.0001 when compared to daf-2; aak(0) using Student’s t-test. (B-C”) The distribution and abundance of activating and repressive chromatin marks are corrected in the daf-2; aak(0); rr166 mutants. The top row (daf-2), middle row (daf-2; aak(0)), and bottom row (daf-2; aak(0); rr166) show (B, B’, B”) H3K4me3 (green), (C, C’, C”) H3K9me3 (green), and DAPI (red). The graphs represent the average immunofluorescence signal of anti-H3K4me3 and anti-H3K9me3 normalized to DAPI in each nucleus across the dissected germ line. All images are merged, condensed Z stacks and are aligned such that distal is left and proximal is right. Due to technical difficulties, only single gonadal arms were analysed (distal, proximal). **P < 0.001 using the F-test for variance when compared to daf-2; aak(0). All animals carry the daf-2(e1370) allele. Scale bar: 4 μm. n = 15. (TIF) [file pgen.1010716.s002.tif]

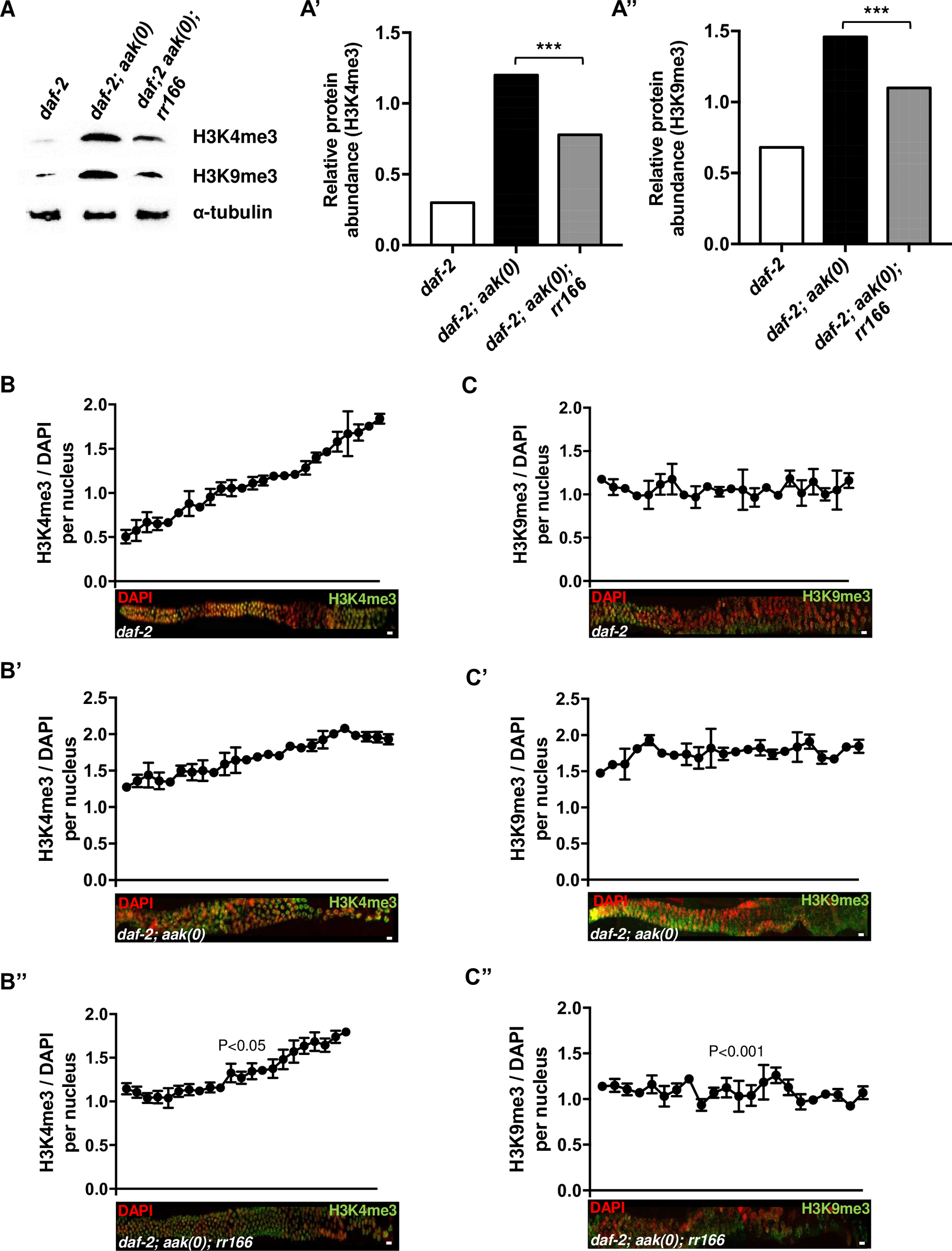

Supplement: S3 Fig — (A) Global levels of H3K4me3 and H3K9me3 were quantified by performing whole-animal western analysis of dauer larvae. (B) Chromatin marks were quantified and normalized to α-tubulin using ImageJ software. ***P < 0.0001 when compared to daf-2; aak(0) using Student’s t-test. (B-C”) The aberrant distribution and abundance of activating (H3K4me3) and repressive (H3K9me3) chromatin marks observed in post-dauer adults that lack AMPK signalling are corrected in the daf-2; aak(0); rr166 mutants. The top row (daf-2), middle row (daf-2; aak(0)), and bottom row (daf-2; aak(0); rr166) show (B, B’, B”) H3K4me3 (green), (C, C’, C”) H3K9me3 (green), and DAPI (red). The graphs represent the average immunofluorescence signal of anti-H3K4me3 and anti-H3K9me3 normalized to DAPI across the dissected germ line. All images are merged, condensed Z stacks that are aligned such that distal is left and proximal is right. Due to technical difficulties, only single gonadal arms were analysed (distal, proximal). **P < 0.001 using the F-test for variance when compared to daf-2; aak(0). All animals carry the daf-2(e1370) allele. Scale bar: 4 μm. n = 15. (TIF) [file pgen.1010716.s003.tif]

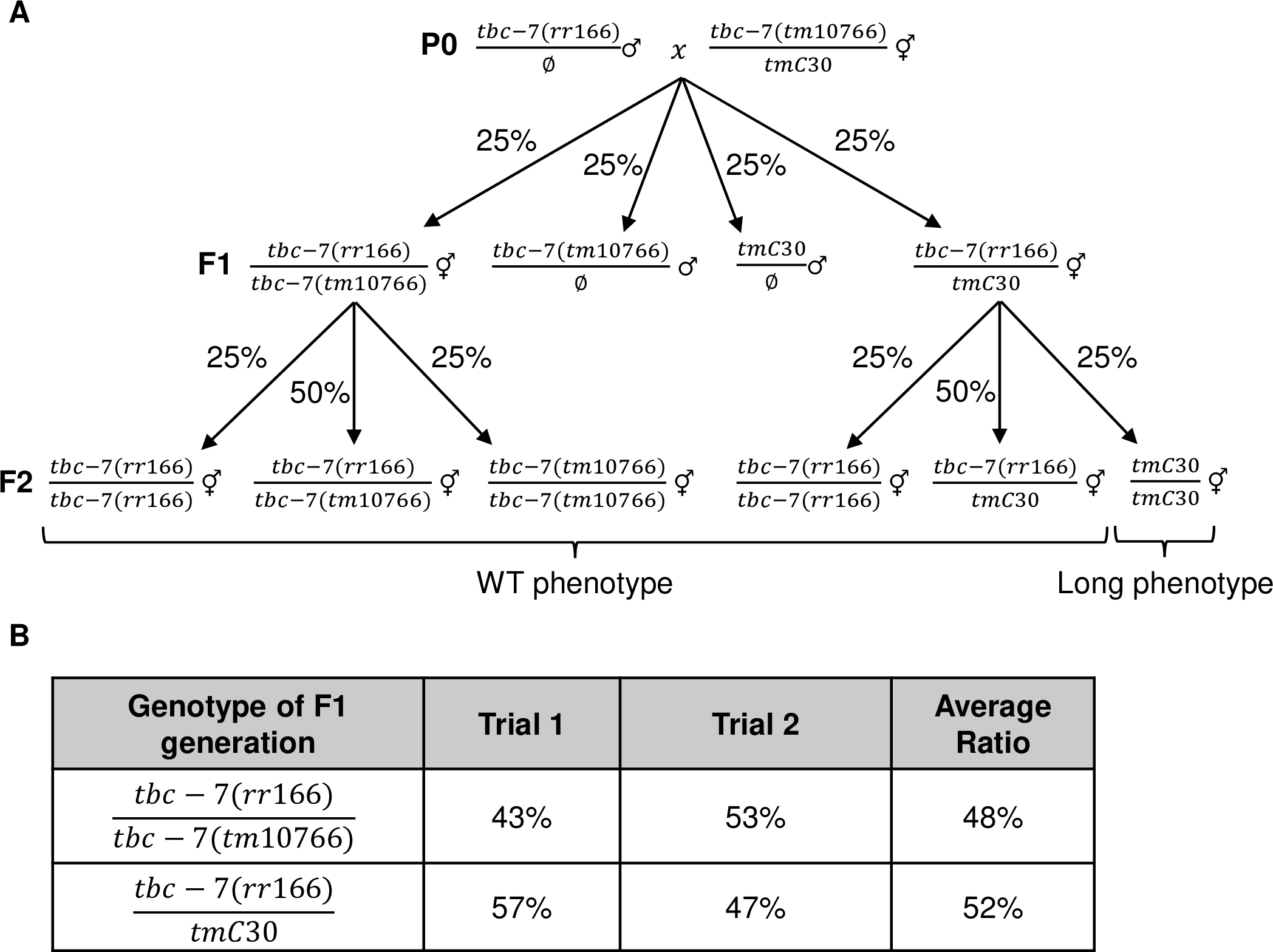

Supplement: S4 Fig — (A) Schematic showing the cross with tbc-7(rr166) with tbc-7(tm10766), which contains a 21,846 bp deletion that completely removes tbc-7. Homozygous tbc-7(tm10766) is non-viable and is balanced with tmC30, which is a balancer that has a recessive Long (Lon) phenotype. To confirm if tbc-7(rr166) is a hypomorphic allele, tbc-7(rr166) was crossed with tbc-7(tm10766) and the F2 cross progeny were examined in order to identify F1 heterozygous tbc-7(rr166)/tbc-7(tm10766) hermaphrodites. F1 heterozygous tbc-7(rr166)/tbc-7(tm10766) hermaphrodites should yield no Lon phenotype progeny, while F1 heterozygous tbc-7(rr166)/tmC30 should yield 25% Lon phenotype progeny. (B) Table showing the percentage of F1 genotypes over two independent crosses. The F2 progeny of every F1 was assessed. F1 genotypes assessed per cross n ≥ 150. (TIF) [file pgen.1010716.s004.tif]

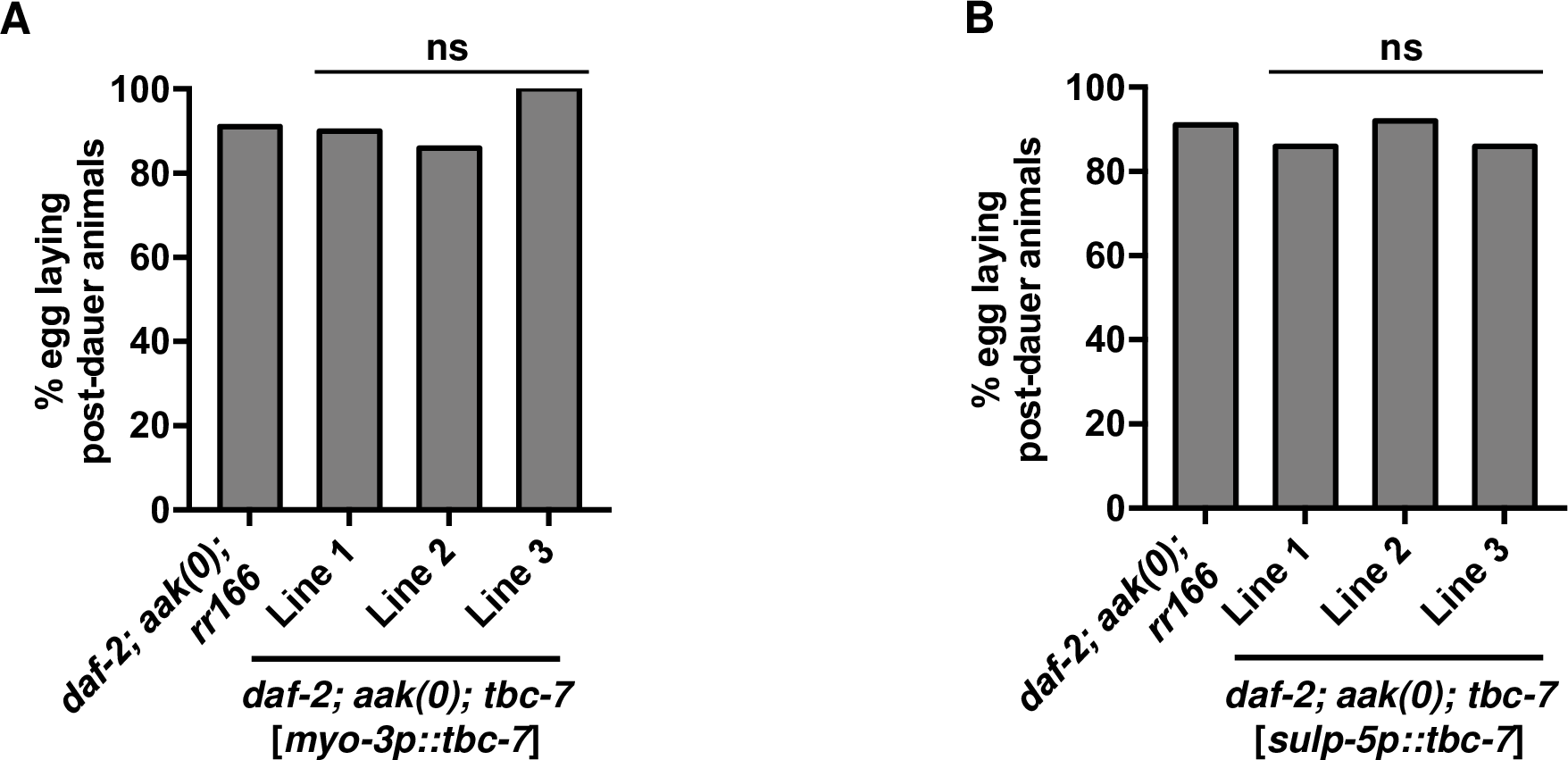

Supplement: S5 Fig — A wild-type copy of tbc-7 cDNA expressed exclusively in the (A) muscles by the myo-3 promoter or the (B) excretory system by the sulp-5 promoter in the tbc-7-suppressed mutants does not revert the suppression of the AMPK germline phenotypes. ns when compared to daf-2; aak(0); tbc-7 based on Marascuilo procedure for % egg laying animals. All animals carry the daf-2(e1370) allele. The values for % egg laying post-dauer animals are presented as means. Each assay was repeated three times with 50 animals in each trial. n = 50. (TIF) [file pgen.1010716.s005.tif]

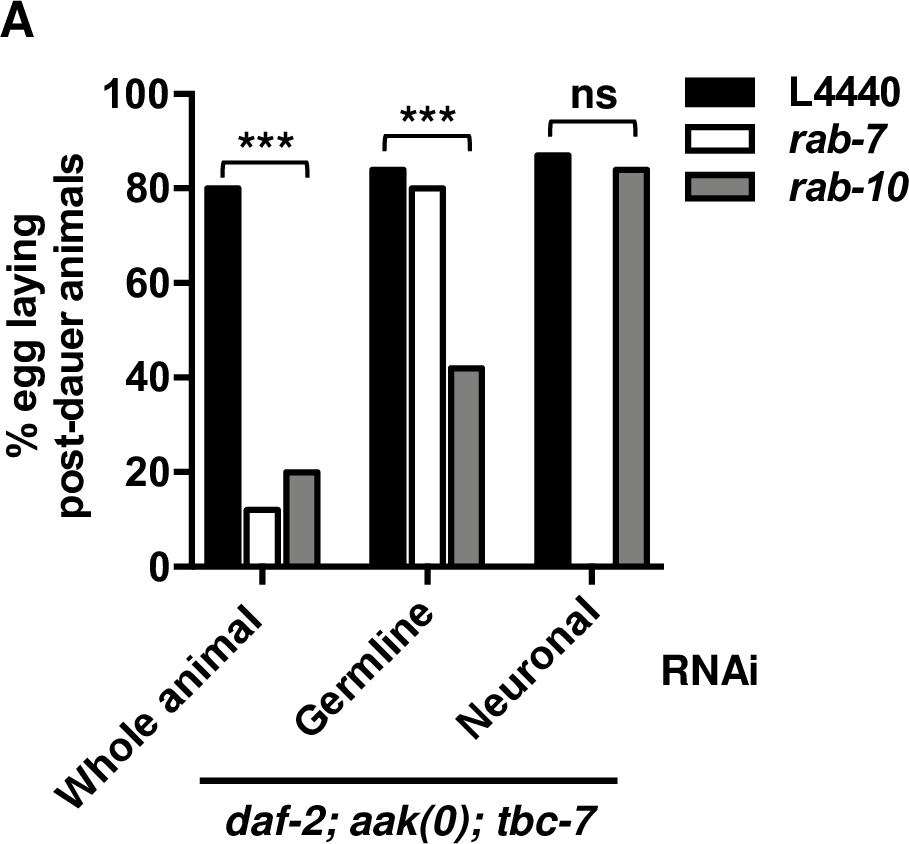

Supplement: S6 Fig — (A) Tissue-specific RNAi experiments in the daf-2; aak(0); tbc-7 mutant show that rab-7 functions in the neurons while rab-10 functions in the germ line. ***P < 0.0001 when compared to L4440 empty vector using Marascuilo procedure for % of egg laying post-dauer animals. The values for % egg laying post-dauer animals are presented as means. Each assay was repeated three times with 50 animals in each trial. n = 50. (TIF) [file pgen.1010716.s006.tif]

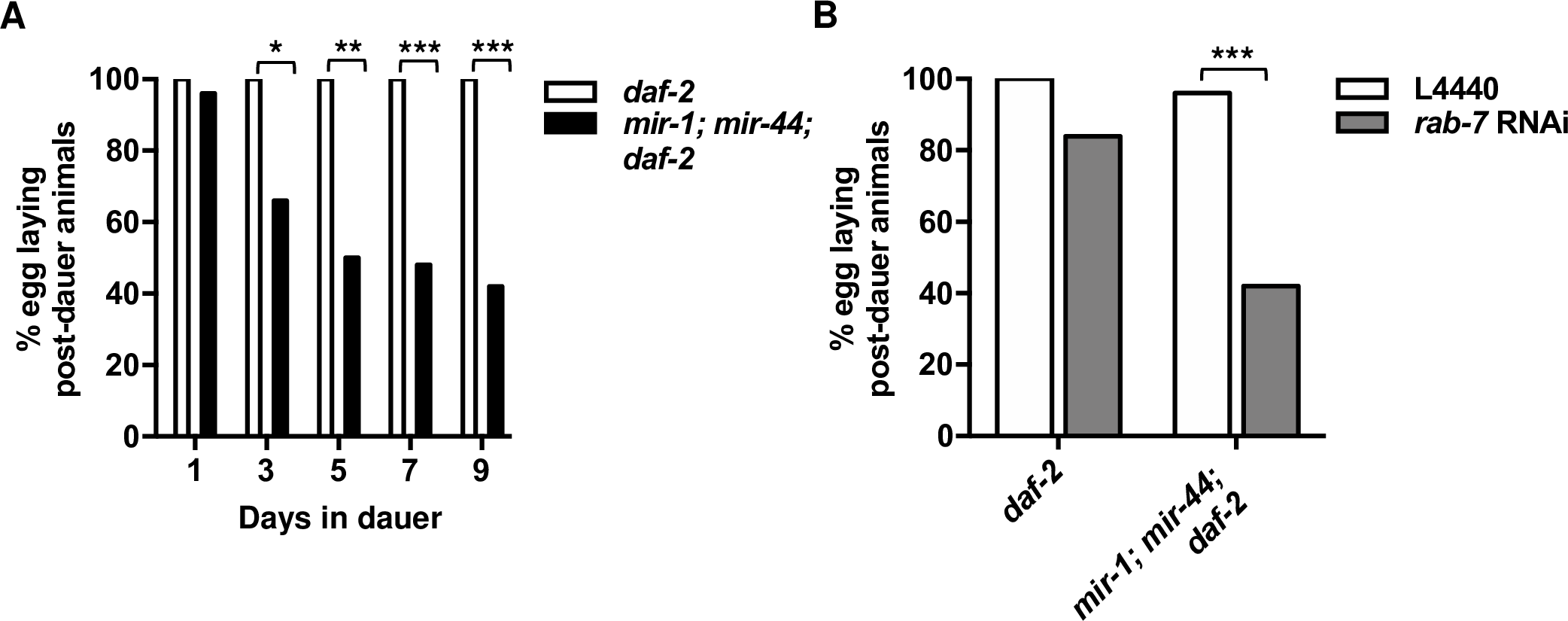

Supplement: S7 Fig — (A) mir-1; mir-44; daf-2 mutants exhibit post-dauer sterility after 7 days in the dauer stage. ***P < 0.0001, **P < 0.001, *P < 0.01 when compared to daf-2 animals that spent an identical duration in the dauer stage using Marascuilo procedure for % egg laying animals. (B) Reducing rab-7 levels greatly enhances/accelerates the post-dauer sterility associated with a loss of mir-1 and mir-44. ***P < 0.0001 when compared to L4440 empty vector using Marascuilo procedure for % egg laying animals. All animals carry the daf-2(e1370) allele. The values for % egg laying post-dauer animals are presented as means. Each assay was repeated three times with 50 animals in each trial. n = 50. (TIF) [file pgen.1010716.s007.tif]

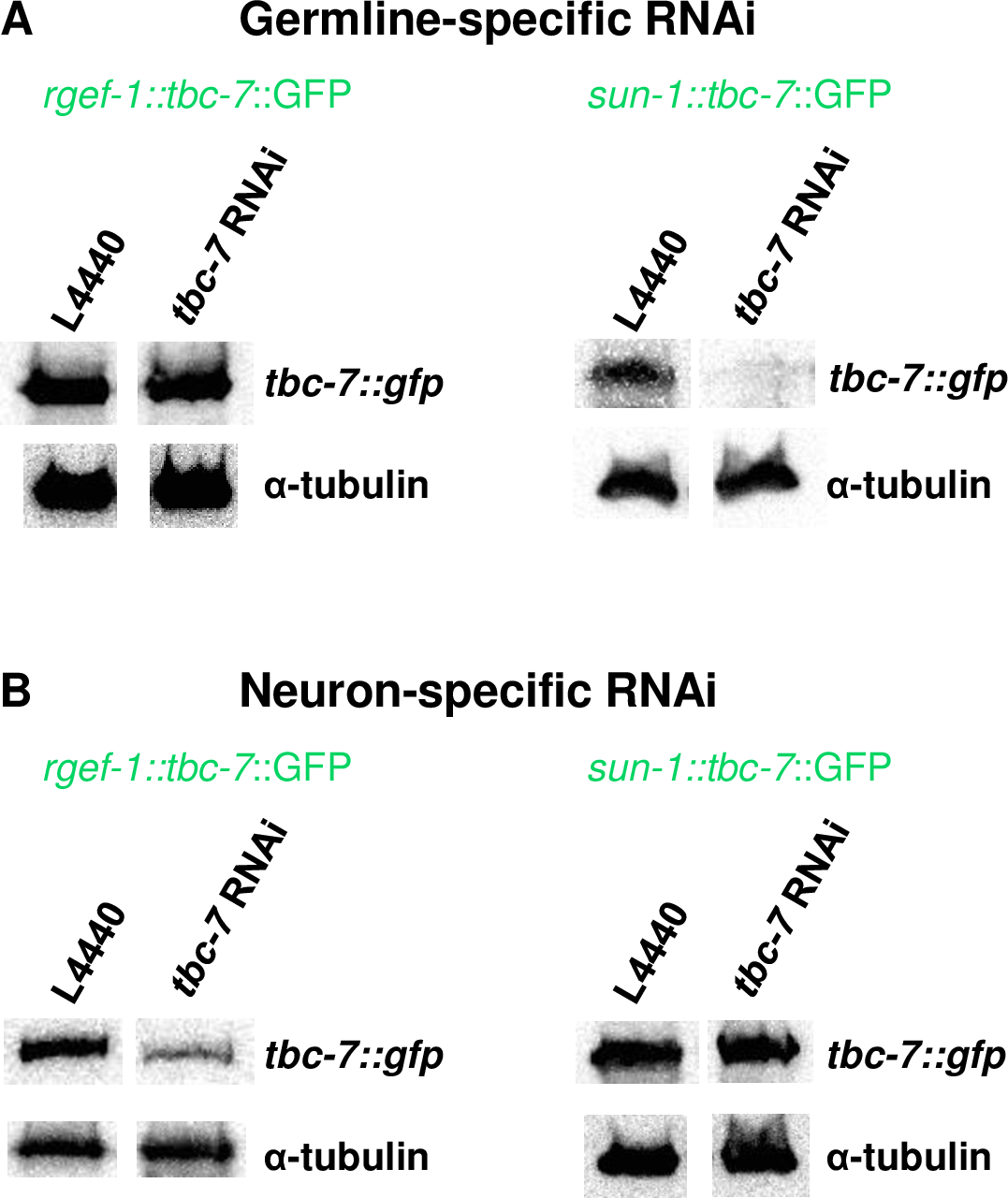

Supplement: S8 Fig — TBC-7::GFP was expressed in either the neurons (rgef-1 promoter) or the in the germ line (sun-1 promoter) of animals with (A) germline-specific RNAi and (B) neuron-specific RNAi. Tissue-specific RNAi animals were treated with either empty vector control L4440 or with dsRNA against tbc-7. The levels of TBC-7::GFP protein expression were quantified using Western blotting against GFP. α-tubulin was used as a loading control. (TIF) [file pgen.1010716.s008.tif]

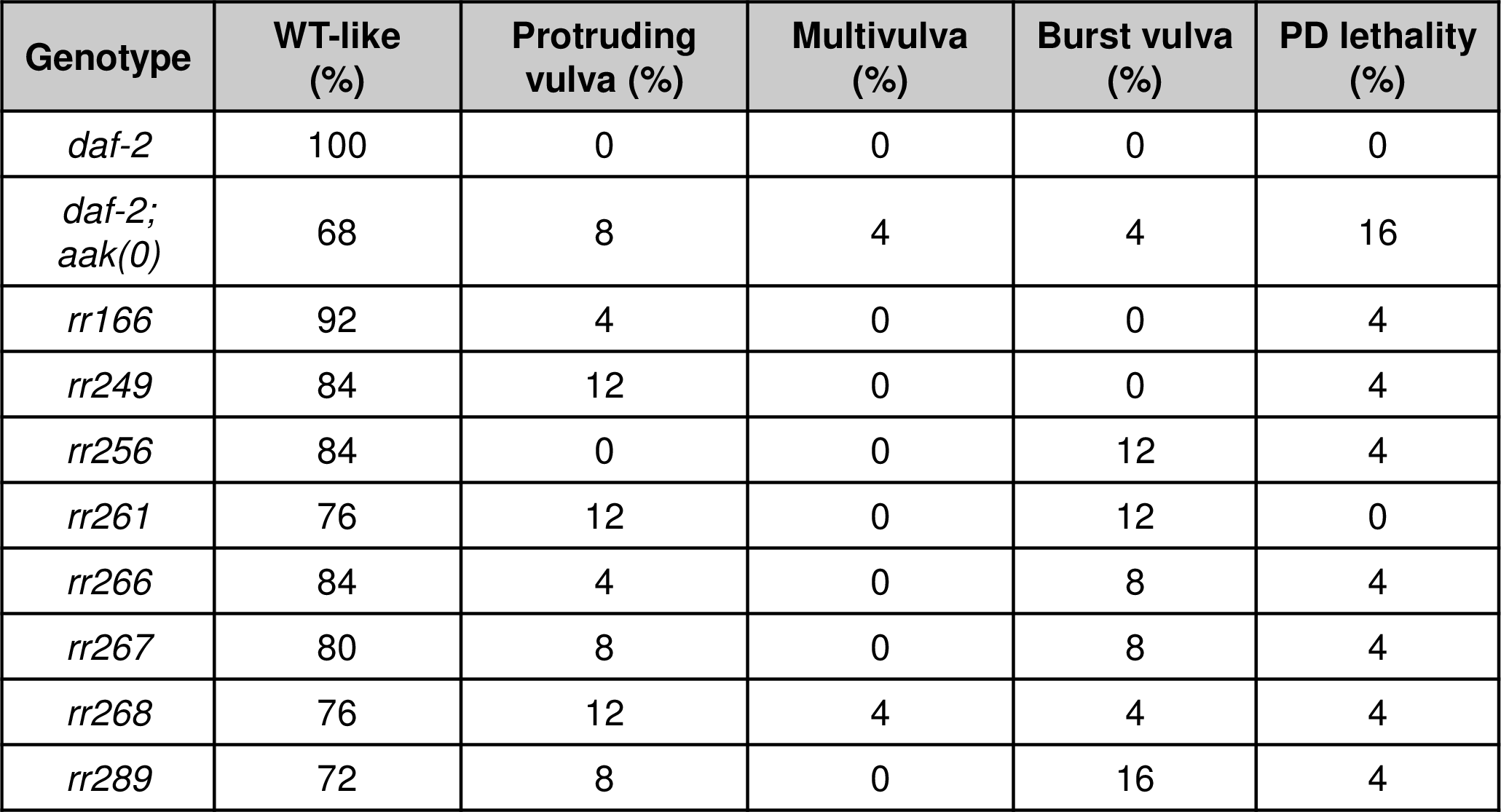

Supplement: S1 Table — Mutants were allowed to transit through the dauer stage and recover. The post-dauer somatic defects were assessed seven days after the recovery period. All animals carry the daf-2(e1370) allele. Mutants isolated from the EMS screen are daf-2; aak(0). The values are presented as means. Each assay was repeated three times with 50 animals in each trial. n = 50. (TIF) [file pgen.1010716.s009.tif]

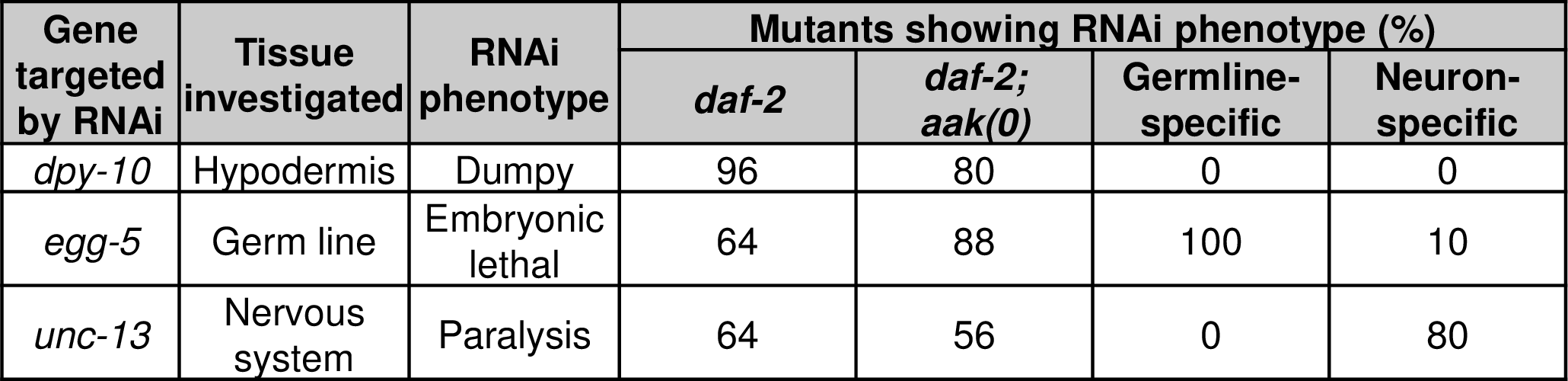

Supplement: S2 Table — To create a tissue-specific RNAi strain in the daf-2; aak(0); rde-1 background, rde-1 was driven exclusively in the neurons using a rgef-1 promoter or in the germ line using a sun-1 promoter to create a neuron-specific or germline-specific RNAi strain, respectively. To confirm that daf-2; aak(0); rde-1 mutants with tissue-specific expression of rde-1 exhibit tissue-specific RNAi phenotypes, mutants were fed dsRNA against dpy-10 (hypodermis), egg-5 (germ line), or unc-13 (neurons) and the phenotypes were scored. Each dsRNA treatment exhibits a unique phenotype, such as dumpy (dpy-10 RNAi), embryonic lethal (egg-5 RNAi), or paralysis (unc-13 RNAi). daf-2 control, daf-2; aak(0), or the tissue-specific RNAi strains were synchronized and plated on bacteria expressing one of three dsRNAs. These animals transited through the dauer stage, and their RNAi phenotype was scored as post-dauer adults. Only mutants expressing rde-1 in the same tissues targeted by the dsRNA should exhibit the RNAi phenotype. All mutants are daf-2; aak(0) except the daf-2 control. The values are presented as means. Each assay was repeated three times with 50 animals in each trial. n = 50. (TIF) [file pgen.1010716.s010.tif]

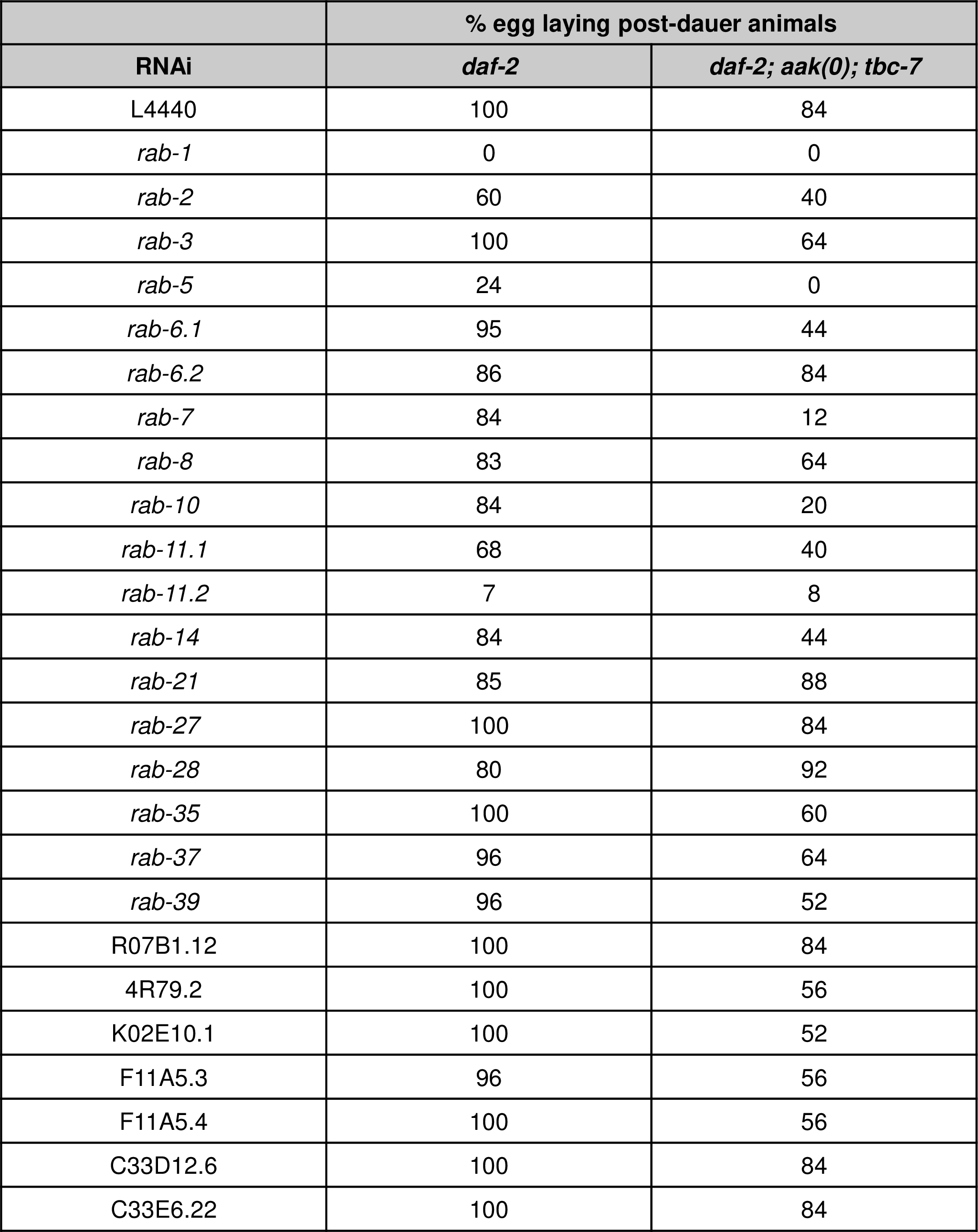

Supplement: S3 Table — daf-2 control and daf-2; aak(0); tbc-7 mutants were fed dsRNA against all known and predicted rab genes then allowed to transit through the dauer stage. L4440 empty vector was used as a control. All animals have the daf-2(e1370) allele. The values for % egg laying post-dauer animals are presented as means. Each assay was repeated three times with 50 animals in each trial. n = 50. (TIF) [file pgen.1010716.s011.tif]

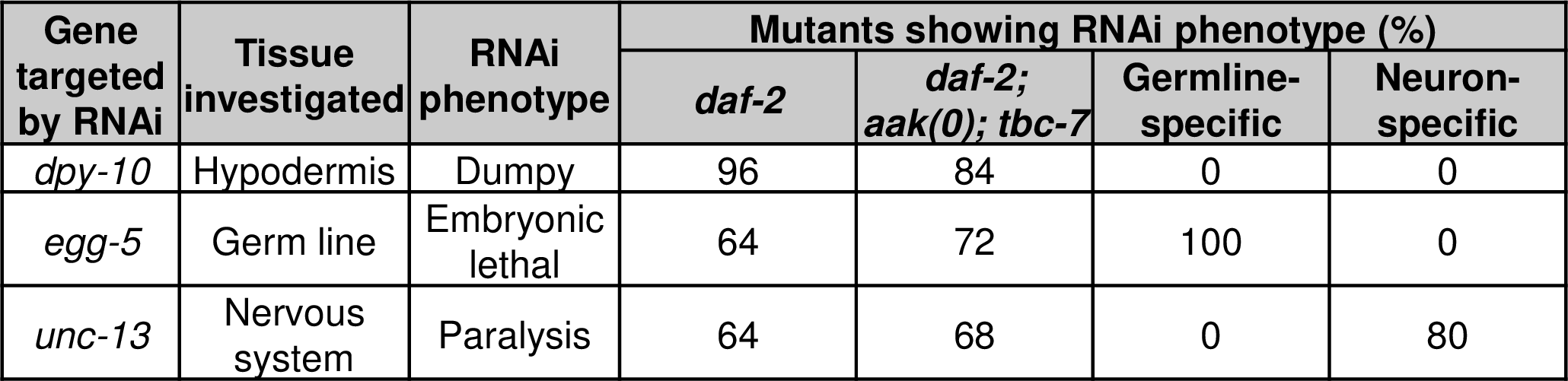

Supplement: S4 Table — To create a tissue-specific RNAi strain in the daf-2; aak(0); rde-1; tbc-7 background, rde-1 was driven exclusively in the neurons using a rgef-1 promoter or in the germ line using a sun-1 promoter to create a neuron-specific or germline-specific RNAi strain, respectively. To confirm that daf-2; aak(0); rde-1; tbc-7 mutants with tissue-specific expression of rde-1 exhibit tissue-specific RNAi phenotypes, mutants were fed dsRNA against dpy-10 (hypodermis), egg-5 (germ line), or unc-13 (neurons) and the phenotypes were scored. Each dsRNA treatment exhibits a unique phenotype, such as dumpy (dpy-10 RNAi), embryonic lethal (egg-5 RNAi), or paralysis (unc-13 RNAi). daf-2 control, daf-2; aak(0), or the tissue-specific RNAi strains were synchronized and plated on bacteria expressing one of three dsRNAs. These animals transited through the dauer stage, and their RNAi phenotype was scored as post-dauer adults. Only mutants expressing rde-1 in the same tissues targeted by the dsRNA should exhibit the RNAi phenotype. All mutants are daf-2; aak(0); tbc-7 except the daf-2 control. The values are presented as means. Each assay was repeated three times with 50 animals in each trial. n = 50. (TIF) [file pgen.1010716.s012.tif]

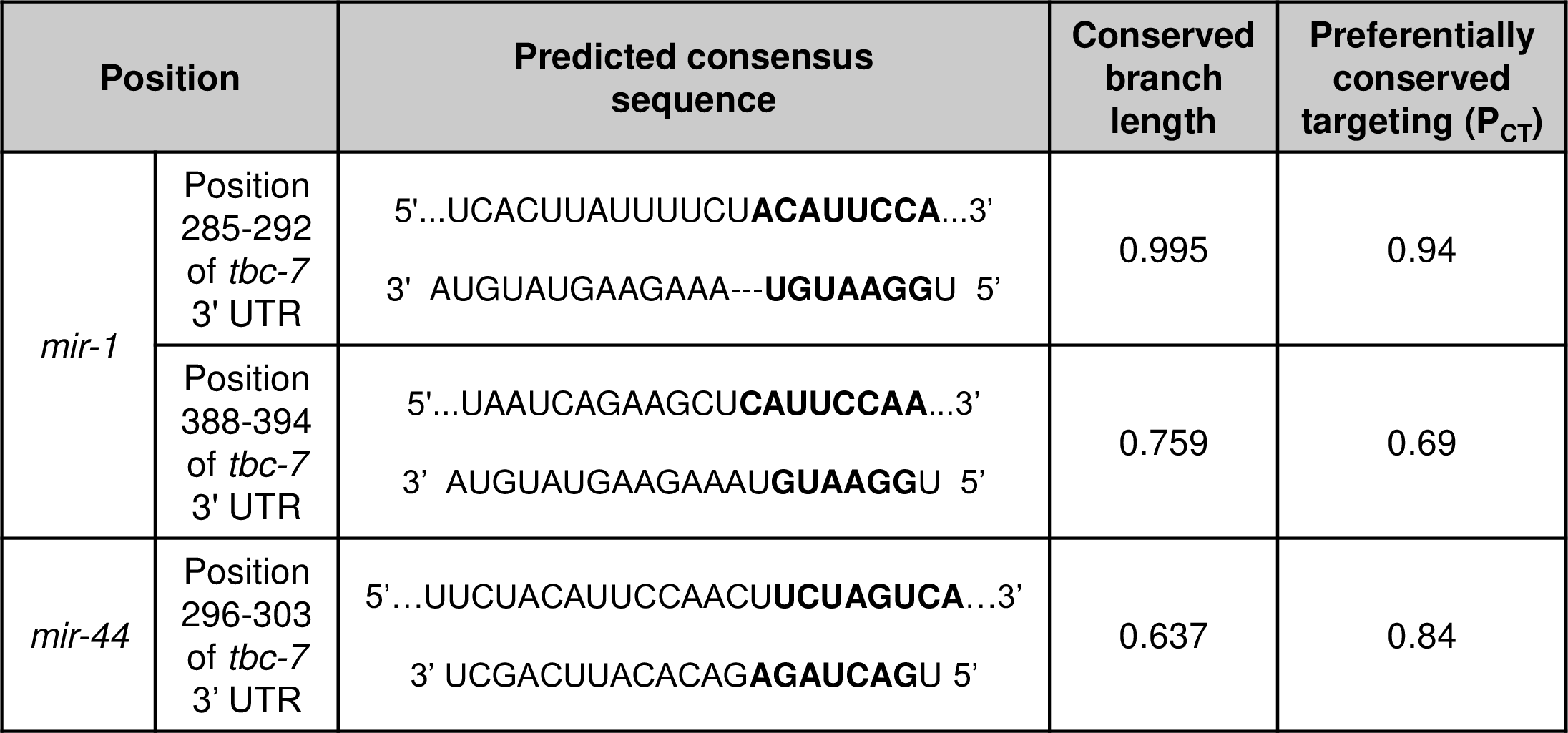

Supplement: S5 Table — Two highly conserved mir-1 seed sequences (bold) and one highly conserved mir-44 seed sequence (bold) were identified in the 3’ UTR of tbc-7 (TargetScanWorm release 6.2), suggesting that mir-1 and mir-44 directly regulates tbc-7 expression. The PCT and conserved branch length are measures of the biological relevance of the predicted miRNA and target interaction, with greater values being more likely to have detectable biological function [50,51]. (TIF) [file pgen.1010716.s013.tif]
