# Supplementary material for: Germline stem cell integrity and quiescence are controlled by an AMPK-dependent neuronal trafficking pathway
Source: PLoS Genet. 2023 Apr 14;19(4):e1010716. doi: 10.1371/journal.pgen.1010716 (PMC10132661; doi:10.1371/journal.pgen.1010716)
Supplement: S6 Table — (DOCX) [file pgen.1010716.s014.docx]

| **Strain** | **Genotype** | **Reference** |
| --- | --- | --- |
| CB1370 | *daf-2(e1370) III* | [1] |
| MR1000 | *daf-2(e1370) aak-1(tm1944) III; aak-2(ok523) X* | [2] |
| MR1963 | *daf-2(e1370) aak-1(tm1944) III; tbc-7(rr166) aak-2(ok523) X* | This study |
| MR2267 | *daf-2(e1370) aak-1(tm1944) III; tbc-7(rr267) aak-2(ok523) X* | This study |
| MR2265 | *daf-2(e1370) aak-1(tm1944) III; rr249 V; aak-2(ok523) X* | This study |
| MR2204 | *daf-2(e1370) aak-1(tm1944) III; rr256 aak-2(ok523) X* | This study |
| MR2205 | *daf-2(e1370) aak-1(tm1944) III; rr266 aak-2(ok523) X* | This study |
| MR2269 | *daf-2(e1370) aak-1(tm1944) III; rr289 aak-2(ok523) X* | This study |
| MR2270 | *daf-2(e1370) aak-1(tm1944) III; rr261 aak-2(ok523) X* | This study |
| MR2268 | *daf-2(e1370) aak-1(tm1944) III; rr268 IV; aak-2(ok523) X* | This study |
| DCL569 | *mkcSi13[sun-1p::rde-1::sun-1 3′UTR + unc-119(+)] II; rde-1(mkc36) V* | [3] |
| MR2292 | *mkcSi13[sun-1p::rde-1::sun-1 3'UTR + unc-119(+)] II; daf-2(e1370) aak-1 (tm1944) III; rde-1(mkc36) V; aak-2(ok524) X* | This study |
| MR2359 | *daf-2(e1370) aak-1(tm1944) III; rde-1(mkc36) V; aak-2(ok524) X* | This study |
| MR2283 | *mkcSi13[sun-1p::rde-1::sun-1 3'UTR + unc-119(+)] II; daf-2(e1370) aak-1(tm1944) III; rde-1(mkc36) V; tbc-7(rr166) aak-2(ok524) X* | This study |
| MR2308 | *daf-2(e1370) aak-1(tm1944) III; rde-1(mkc36) V; tbc-7(rr166) aak-2(ok524) X* | This study |
| VC576 | *mir-1(gk276) I* | *C. elegans* Reverse Genetics Core Facility, UBC |
| MR2289 | *mir-1(gk276) I; daf-2(e1370) III* | This study |
| MT17431 | *nDf49[mir-44; ZK930.12; mir-42; ZK930.15; mir-43] II; nDf59[mir-61; mir-250; sel-11] V; mir-247(n4505) X* | [4] |
| MR2592 | *nDf49[mir-44; ZK930.12; mir-42; ZK930.15; mir-43] II; daf-2(e1370) III* | This study |
| MR2593 | *mir-1(gk276) I; nDf49[mir-44; ZK930.12; mir-42; ZK930.15; mir-43] II; daf-2(e1370) III* | This study |
| MR2324 | *tbc-7(rr166) X* | This study |
| FX31259 | *tbc-7(tm10766)/tmC30(tmIS1247) X* | National Bioresource Project *C. elegans*, Toyko, Japan |
| MR2653 | *daf-2(e1370) aak-1(tm1944) III; tbc-7(rr166) aak-2(ok523) X; rrEx688[fosmid tbc-7::GFP; rol-6] line 1* | This study |
| MR2654 | *daf-2(e1370) aak-1(tm1944) III; tbc-7(rr166) aak-2(ok523) X; rrEx689[fosmid tbc-7::GFP; rol-6] line 2* | This study |
| MR2655 | *daf-2(e1370) aak-1(tm1944) III; tbc-7(rr166) aak-2(ok523) X; rrEx690[fosmid tbc-7::GFP; rol-6] line 3* | This study |
| MR2325 | *daf-2(e1370) aak-1(tm1944) III; tbc-7(rr166) aak-2(ok523) X; rrEx562[unc-119::tbc-7::GFP; rol-6] line 1* | This study |
| MR2326 | *daf-2(e1370) aak-1(tm1944) III; tbc-7(rr166) aak-2(ok523) X; rrEx563[unc-119::tbc-7::GFP; rol-6] line 2* | This study |
| MR2327 | *daf-2(e1370) aak-1(tm1944) III; tbc-7(rr166) aak-2(ok523) X; rrEx564[unc-119::tbc-7::GFP; rol-6] line 3* | This study |
| MR2439 | *daf-2(e1370) aak-1(tm1944) III; rde-1(mkc36) V; aak-2(ok523) X; rrEx521[rgef-1::sid-1; rgef-1::rde-1; rol-6]* | This study |
| MR2337 | *daf-2(e1370) aak-1(tm1944) III; rde-1(mkc36) V; tbc-7(rr166) aak-2(ok523) X; rrEx527[[rgef-1::sid-1; rgef-1::rde-1; rol-6]* | This study |
| MR2428 | *daf-2(e1370) aak-1(tm1944) III; aak-2(ok523) X; rrEx555[rgef-1::GFP::rab-7; rol-6] line 1* | This study |
| MR2429 | *daf-2(e1370) aak-1(tm1944) III; aak-2(ok523) X; rrEx556[rgef-1::GFP::rab-7; rol-6] line 2* | This study |
| MR2430 | *daf-2(e1370) aak-1(tm1944) III; aak-2(ok523) X; rrEx557[rgef-1::GFP::rab-7; rol-6] line 3* | This study |
| MR2431 | *daf-2(e1370) aak-1(tm1944) III; aak-2(ok523) X; rrEx557[rgef-1::GFP::rab-7 GTP-locked; rol-6] line 1* | This study |
| MR2432 | *daf-2(e1370) aak-1(tm1944) III; aak-2(ok523) X; rrEx558[rgef-1::GFP::rab-7 GTP-locked; rol-6] line 2* | This study |
| MR2433 | *daf-2(e1370) aak-1(tm1944) III; aak-2(ok523) X; rrEx559[rgef-1::GFP::rab-7 GTP-locked; rol-6] line 3* | This study |
| MR2522 | *daf-2(e1370) III; rrEx594[rgef-1::tbc-7::3’UTR deletion; rol-6] line 1* | This study |
| MR2523 | *daf-2(e1370) III; rrEx595[rgef-1::tbc-7::3’UTR deletion; rol-6] line 2* | This study |
| MR2524 | *daf-2(e1370) III; rrEx596[rgef-1::tbc-7::3’UTR deletion; rol-6] line 3* | This study |
| MR2529 | *daf-2(e1370) III; rrEx601[rgef-1::tbc-7::3’UTR wild type; rol-6] line 1* | This study |
| MR2530 | *daf-2(e1370) III; rrEx602[rgef-1::tbc-7::3’UTR wild type; rol-6] line 2* | This study |
| MR2532 | *daf-2(e1370) III; rrEx603[rgef-1::tbc-7::3’UTR wild type; rol-6] line 3* | This study |
| MR2638 | *daf-2(e1370) aak-1(tm1944) III; aak-2(ok523) X; rrEx673[rgef-1::mir-1; rol-6] line 1* | This study |
| MR2639 | *daf-2(e1370) aak-1(tm1944) III; aak-2(ok523) X; rrEx674[rgef-1::mir-1; rol-6] line 2* | This study |
| MR2640 | *daf-2(e1370) aak-1(tm1944) III; aak-2(ok523) X; rrEx675[rgef-1::mir-1; rol-6] line 3* | This study |
| MR2680 | *daf-2(e1370) aak-1(tm1944) III; aak-2(ok523) X; rrEx714[rgef-1::mir-44; rol-6] line 1* | This study |
| MR2681 | *daf-2(e1370) aak-1(tm1944) III; aak-2(ok523) X; rrEx715[rgef-1::mir-44; rol-6] line 2* | This study |
| MR2682 | *daf-2(e1370) aak-1(tm1944) III; aak-2(ok523) X; rrEx716[rgef-1::mir-44; rol-6] line 3* | This study |
| MR2600 | *daf-2(e1370) III; rrEx720[rgef-1::tbc-7::GFP; rol-6] line 1* | This study |
| MR2601 | *daf-2(e1370) aak-1(tm1944) III; rrEx721[rgef-1::tbc-7::GFP; rol-6] line 1* | This study |
| MR2448 | *daf-2(e1370) III; rrEx530[rgef-1::tbc-7 S115A; rol-6] line 1* | This study |
| MR2449 | *daf-2(e1370) III; rrEx531[rgef-1::tbc-7 S115A; rol-6] line 2* | This study |
| MR2450 | *daf-2(e1370) III; rrEx532[rgef-1::tbc-7 S115A; rol-6] line 3* | This study |
| MR2663 | *daf-2(e1370) aak-1(tm1944) III; aak-2(ok524) X; rrEx597[rgef-1::tbc-7 S115E; rol-6] line 1* | This study |
| MR2662 | *daf-2(e1370) aak-1(tm1944) III; aak-2(ok524) X; rrEx598[rgef-1::tbc-7 S115E; rol-6] line 2* | This study |
| MR2665 | *daf-2(e1370) aak-1(tm1944) III; aak-2(ok524) X; rrEx599[rgef-1::tbc-7 S115E; rol-6] line 3* | This study |
| MR2656 | *daf-2(e1370) aak-1(tm1944) III; tbc-7(rr166) aak-2(ok523) X; rrEx691[myo-3::tbc-7; rol-6] line 1* | This study |
| MR2657 | *daf-2(e1370) aak-1(tm1944) III; tbc-7(rr166) aak-2(ok523) X; rrEx692[myo-3::tbc-7; rol-6] line 2* | This study |
| MR2658 | *daf-2(e1370) aak-1(tm1944) III; tbc-7(rr166) aak-2(ok523) X; rrEx693[myo-3::tbc-7; rol-6] line 3* | This study |
| MR2739 | *daf-2(e1370) aak-1(tm1944) III; tbc-7(rr166) aak-2(ok523) X; rrEx766[sulp-5::tbc-7; rol-6] line 1* | This study |
| MR2741 | *daf-2(e1370) aak-1(tm1944) III; tbc-7(rr166) aak-2(ok523) X; rrEx768[sulp-5::tbc-7; rol-6] line 2* | This study |
| MR2745 | *daf-2(e1370) aak-1(tm1944) III; tbc-7(rr166) aak-2(ok523) X; rrEx772[sulp-5::tbc-7; rol-6] line 3* | This study |
| MR2632 | *daf-2(e1370) III; rrEx667[rgef-1::HA::tbc-7 S520F; rol-6] line 1* | This study |
| MR2633 | *daf-2(e1370) III; rde-1(mkc36) V; rrEx668[rgef-1::sid-1; rgef-1::rde-1; rgef-1::tbc-7::GFP; rol-6] line 1* | This study |
| MR2673 | *mkcSi13[sun-1p::rde-1::sun-1 3'UTR + unc-119(+)] II; daf-2(e1370) III; rde-1(mkc36) V; rrEx707[rgef-1::tbc-7::GFP; rol-6] line 1* | This study |

**Table S6: A list of strains used in this study.**

**References**

1. Kimura KD, Tissenbaum HA, Liu Y, Ruvkun G. daf-2, an insulin receptor-like gene that regulates longevity and diapause in Caenorhabditis elegans. Science (New York, NY). 1997;277(5328):942-6.

2. Kadekar P, Roy R. AMPK regulates germline stem cell quiescence and integrity through an endogenous small RNA pathway. PLoS Biol. 2019;17(6):e3000309.

3. Zou L, Wu D, Zang X, Wang Z, Wu Z, Chen D. Construction of a germline-specific RNAi tool in C. elegans. Sci Rep. 2019;9(1):2354.

4. Alvarez-Saavedra E, Horvitz HR. Many families of C. elegans microRNAs are not essential for development or viability. Current biology : CB. 2010;20(4):367-73.
