# Supplementary material for: Germline stem cell integrity and quiescence are controlled by an AMPK-dependent neuronal trafficking pathway
Source: PLoS Genet. 2023 Apr 14;19(4):e1010716. doi: 10.1371/journal.pgen.1010716 (PMC10132661; doi:10.1371/journal.pgen.1010716)
Supplement: Attachment — Submitted filename: Response to PLOS Biology Reviewers Comments.pdf [file pgen.1010716.s016.pdf]

PLOS Biology Reviewers' Comments

Reviewer 1

In this study, the authors seek to identify new regulators of germline quiescence during *C. elegans* dauer stage (an arrested development larval stage induced by environmental stress).

Signaling from the AMP-activated protein kinase (AMPK) homolog in the worm, aak-1, is required for arrest of germline stem cells at this stage, with null mutations causing germline hyperplasia.

To find new regulators, the authors perform a forward genetics screen for suppressors of germline hyperplasia caused by null mutation of aak-1. The authors show that:

1. Eight alleles, forming 5 complementation groups, suppress the aak-1 mutant phenotype. The authors chose to focus their work on the allele having the biggest effect (*rr166*).
2. aak-1 null animals present chromatin defects at the dauer and post-dauer stages, which are corrected when *rr166* is also present.
3. *rr166* is a G->A transition mutation in the gene *tbc-7*, a RabGAP protein that is expressed in neurons and antagonizes AMPK.
4. Numerous RABs could be regulated by *tbc-7*. In particular, the authors focus on *rab-7*, which they show is required in neurons (in its GTP bound form) to repress the defects caused by aak-1 loss of function.
5. Involvement of *mir-1*.
6. Phosphorylation of Ser115 of *tbc-7*, presumably by AMPK, prevents germline hyperplasia and post-dauer sterility.

Major concerns

* The authors introduce their work highlighting the involvement of small RNA pathways in regulation of AMPK signaling. However, this previous work (from the same lab) implicated an endogenous small RNA pathway that is specifically active in the *C. elegans* germline (the 26G pathway) but provided no evidence that points directly to miRNA involvement. There are multiple small RNA pathways in *C. elegans*, with independent functions. Making the connection between the previous work implicating the 26G RNAs with the possible involvement of a miRNA in this case, is inaccurate and misleading.

In Kadekar et al, PLOS Biology 2019, we showed that by disabling some of the endogenous siRNA pathways, we could suppress the AMPK germline defects, suggesting that the production of siRNAs is maladaptive to the animal during the dauer stage. Our attempts were not comprehensive, and later we found that CSR-1 is also involved in dauer development through some unknown mechanism, but we did not

know this at the outset. However, we did not mean to imply that microRNAs are dispensable in the maintenance of germ cell integrity while entering the dauer stage. In fact, microRNA production is critical for entering the dauer stage and genetic mutants that are deficient in producing microRNAs are also dauer-deficient and cannot enter the dauer stage. Thus, when discussing the dauer stage, it is implied that microRNAs are involved. It requires more directed approaches to assess the role of these important small RNAs in the context of dauer development due to their essential role in this developmental stage. In this manuscript, we highlighted the requirement of at least two microRNAs, *mir-1* and *mir-44* in the regulation of *tbc-7*. While these two microRNAs are not involved in dauer entry, our data suggest that they directly downregulate *tbc-7* activity in order to maintain RAB-7 in its active GTP-bound form.

* When the authors determine that *rr166* is an allele of *tbc-7*, they do so by showing that RNAi against *tbc-7* has a similar phenotype to *rr166* but they also attempt to rescue the *rr166* phenotype by injecting wild-type *tbc-7* DNA. The effect of this rescue is really not very convincing. The authors show a single transgenic line that shows a small amount of rescue. How this line was generated is not explained at all (in fact there is no information in the methods about any of the multiple transgenic lines in this study - the authors need to include what type of transgenes they use, what DNA was injected, at what concentration). Is over-expression of *tbc-7* perhaps also detrimental?

In this study, we used a fosmid that contains a wild type copy of *tbc-7* to show that *rr166* is an allele of *tbc-7*. Fosmids are advantageous over other methods because they contain large genomic regions that are more likely to capture most, if not all, cis-regulatory information, therefore providing a more accurate representation of the expression of any given gene in addition to the full length of this gene. Three independently generated mutant lines of *daf-2; aak(0); tbc-7* animals carrying this fosmid as an extrachromosomal array were generated, and results obtained with those lines were added to the manuscript to replace the cosmid data, as suggested by the reviewer. These changes are reflected in Fig 2A and in the text on lines 236-239.

Further experiments were included to support the claim that *rr166* is an allele of *tbc-7*. First, we used a known null allele of *tbc-7(tm10766)* isolated by the NRBP in Japan. The deletion of *tbc-7* is lethal; thus *tbc-7(tm10766)* cannot be maintained as homozygous mutants. By crossing our isolated *tbc-7*(*rr166*) allele with the *tbc-7(tm10766)* null allele, we were able to isolate heterozygous *tbc-7(rr166)/tbc-7(tm10766)* mutants, suggesting our that our allele of *tbc-7*(*rr166*) is a hypomorphic allele of *tbc-7* and is sufficient to generate viable animals. This experiment is described in S4 Fig and in the text on lines 257-262.

Furthermore, when a wild type copy of *tbc-7* was expressed in the neurons of *daf-2*; *aak(0)*; *tbc-7* mutants using a pan-neuronal promoter, the suppression of AMPK germline defects was reversed, indicating that the EMS-induced lesion present in the *tbc-7(rr166)* is certainly linked to a loss of TBC-7 function. Furthermore, the *tbc-7* function that affects the germ cells of AMPK mutant dauer larvae must reside in the

nervous system. This experiment is shown in Fig 2D and is described in the text on lines 284-287.

Details and information regarding the generation of all genetic mutants and transgenic strains can be found in the updated Methods and Materials section (in the text lines 671-686). The specific concentrations of the fosmid injections can be found in the same section.

We have not examined at which concentration of exogenously injected *tbc-7* would cause control *daf-2* animals to suffer from germline defects. Injecting a plasmid driving *tbc-7* in the neurons at 15 ng/μL does not negatively affect the post-dauer fertility of control *daf-2* animals; *daf-2* animals expressing extra copies of neuronal wild-type *tbc-7* have 100% post-dauer fertility (as shown in Fig 4D and Fig 5D).

* Also, the authors never state what type of effect *rr166* has at the protein level. Is it missense or non-sense mutation, for example? The fact that they can isolate *rr166* over a deletion of *tbc-7* suggests it is a very weak hypomorph and yet it has a dramatic effect on the germline - but this cannot be easily rescued (as brought up in the point above). If *tbc-7* is so dose sensitive, one might expect that the balanced heterozygote carrying the *tbc-7* deletion *(tm10766)* should also have a phenotype in the assays shown in Fig. 1. And since the authors can isolate *rr166*/tm10766 animals, it would be interesting to analyze those animals as well.

The EMS-induced lesion of *rr166* causes a missense mutation from serine on residue 520 to phenylalanine (S520F). To examine if the S520F mutation affects the protein levels of TBC-7, we conducted a Western blot against wild-type TBC-7 and TBC-7 S520F in dauer larvae and adult animals. Western analysis shows that the S520F mutation does not affect the stability or the expression of TBC-7 at either of these life stages, suggesting that perhaps the S520F disables the catalytic activity of TBC-7. These data are currently missing from our submission but will be included should it be accepted for review. The western analysis is attached below:

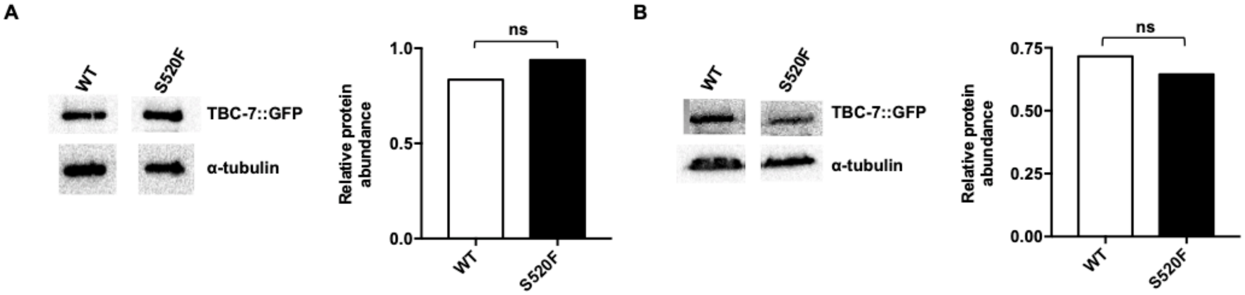

S4 Fig. EMS-induced S520F mutation does not affect the stability or the expression of TBC-7. A wild type or S520F variant of TBC-7 translational fused with GFP was expressed under the native *tbc-7* promoter (2026 bp upstream of the transcription start site). Western analysis was conducted using an antibody raised against GFP shows that the stability or expression of TBC-7 is not affected by the S520F mutation in (A) dauer larvae or (B) adult animals. TBC-7 expression was quantified and normalized to α-tubulin using ImageJ software. ns when compared to control using Student's t-test.

We were able to isolate a heterozygous *tbc-7(rr166)/tbc-7(tm10766)* mutant in the wild-type N2 background, which indicates that our *tbc-7(rr166)* allele is a hypomorphic allele. Although we did not examine the post-dauer fertility and germ cell count of *daf-2; aak(0); tbc-7(rr166)/tbc-7(tm10766)* mutants, we assume that these mutants would be able to suppress the AMPK germline defects given that *tbc-7* is inactivated/not expressed, allowing RAB-7 to remain in its active GTP-bound form. Isolating this quadruple mutant would be very difficult given that *aak-2* and *tbc-7* are on the same X chromosome.

* To study the tissue of action of *tbc-7* the authors use both tissue-specific re-introduction of and tissue-specific knock down of *tbc-7*. But for both these experiments, they only look at neuronal expression/knockdown. It would help interpret results if we knew whether other somatic tissues also contribute. The specificity and efficiency of the neuron specific RNAi strain should also be tested, this can be easily done with fluorescent reporters expressed in neurons or muscles and feeding RNAi against the reporter.

To address whether other somatic tissues contribute to the suppression of AMPK germline defects through a *tbc-7*-dependent manner, we expressed *tbc-7* in the muscles (through a *myo-3* promoter) and in the excretory system (through a *sulp-5* promoter) in *daf-2; aak(0); tbc-7* mutants. We did not observe any effects on the post-dauer fertility of the *tbc-7* suppressed mutants when wild-type copies of *tbc-7* were reintroduced in these tissues using these promoters, suggesting that *tbc-7* can only regulate germ cell integrity when expressed in the neurons during the dauer stage. These experiments can be found in S5 Fig and in the text on lines 290-306.

To ensure the specificity and efficiency of the neuron- and germline-specific RNAi strains, we conducted phenotypic assays as well as a series of Western analyses. In our phenotypic assays, mutants were fed dsRNA against *dpy-10* (hypodermis), *egg-5* (germ line), or *unc-13* (neurons) and the phenotypes were scored. Only mutants expressing *rde-1* in the same tissues targeted by the dsRNA exhibited the RNAi phenotype. While the penetrance of the RNAi-induced phenotype was not 100%, we were able to observe a high level of specificity (eg. neuron-specific RNAi mutants did not show dumpy phenotypes when treated with dsRNA against *dpy-10*, which is a hypodermis-specific gene). These assays were done in the *aak(0)* and *aak(0); tbc-7* genetic backgrounds. These experiments can be found in S2 Table, S4 Table, and in the text lines 744-758.

We also demonstrated the specificities of these RNAi-specific strains through a tissue-specific TBC-7::GFP sensor in the neurons and the germ line. TBC-7::GFP was expressed in either the neurons (*rgef-1p*) or the germ line (*sun-1p*) in animals with either neuron- or germline-specific RNAi. These transgenic animals were then treated with either a control empty vector L4440 or dsRNA against *tbc-7*. The levels of TBC-7::GFP were then quantified by Western analysis with an antibody raised against GFP. Only when animals were treated with dsRNA against *tbc-7* where TBC-7::GFP is expressed in the same tissue where RNAi is enabled, did we observe a reduction in the levels of

TBC-7::GFP. Furthermore, the levels of TBC-7::GFP do not change in other tissues that are not targeted with RNAi, suggesting that our RNAi-specific strains have a high level of tissue-specificity and show little to no “leaky” effects due to spreading. These experiments are shown in S7 Fig and are described in the text on lines 758-761.

* For many experiments, the authors show mutants or RNAi treatments and "control" conditions. What the controls are is not stated typically in the legends or the text. This is important as for some experiments, multiple controls are possible. I cannot assess the results without this information. For example in Figure 5B, we assume the control is wt. If this is the case, and the author's model is correct, *rab-7* knockdown in a wt background should also cause an egg laying defect, as *rab-7* is downstream of aak-1 and *tbc-7*.

In response to the reviewer’s suggestion, the full genotypes of all the controls were added in every figure legend. Adding the full genotype of every strain in the graph would clutter the figures.

The last point about *rab-7* RNAi in the *daf-2* background is hard to address. I believe that the animal has many pathways to preserve germ cell integrity during the dauer stage (eg. compromising endogenous siRNA pathway, compromising *tbc-7* expression), thus compromising *rab-7* in a wild type background won’t render the post-dauer animal completely sterile as there are other redundant pathways. In addition, the post-dauer fertility of the *daf-2* control animals after *rab-7* RNAi treatment is reduced to 82% from 100%, indicating that the compromise of *rab-7* caused some egg-laying defect. There are clearly other RAB-7-dependent processes that are not involved in this phenomenon, so we use RNAi to find that sweet spot that allows us to dissect this dauer-associated function without killing the animal by disabling its other essential function(s).

* *mir-1* is a very well-known muscle-specific miRNA. There is no evidence (in the literature or in this work) that shows *mir-1* expression or function in neurons, particularly in *C. elegans*. The authors also fail to take into account two studies in *C. elegans* that report on the contribution of *tbc-7* as a target of *mir-1* (Nehammer et al. eLife 2019. Gutierrez Perez et al Sci Adv 2021).

While is there is no published evidence for *mir-1* functioning in the neurons in *C. elegans*, *mir-1* has not been studied in the context of the dauer larvae. Perhaps in the dauer stage, *mir-1* could act in the neurons in a cell nonautonomous manner to regulate its targets. In this study, we show that *mir-1* deletion mutants eventually exhibit a loss of post-dauer fertility suggesting that *tbc-7* might be misregulated in the absence of *mir-1* (in Fig 4B). In the Nehammer et al eLife 2019 study, a transcriptional reporter using the promoter of *tbc-7* showed GFP expression in the head and tail ganglia in addition to BWM expression (Nehammer et al eLife 2019 Fig S2), supporting our finding that *tbc-7* could be regulated by *mir-1* in the neurons. In addition, we also show that the overexpression of *mir-1* in the neurons of *aak(0)* mutants can suppress the AMPK germline defects. These data suggest that *mir-1* has the capability of regulating *tbc-7*

expression in the neurons, albeit with the caveat that an overexpression study is not indicative of what occurs in normal physiological conditions.

* The defects shown upon mir-1 deletion can be caused by deregulation of targets occurring in other tissues (mainly muscles / pharynx). The authors show no direct evidence that mir-1 is regulating tbc-7 / rab-7 in neurons (reporters, etc.). Animals lacking mir-1 have several defects, including autophagy defects (Nehammer et al.) which are likely synthetic with knockdown of rab-7 regardless of the phenomenon that is the focus of this study.

mir-1 has been well studied in the aspect of regulating autophagy and protein aggregation. However, no studies have reported a loss of fertility in either the dauer stage or development in replete conditions due to a loss of *mir-1*. Furthermore, the modification of the potential *mir-1* and *mir-44* binding sites in the 3'UTR of *tbc-7* result in a clear phenotype. This is perhaps not direct enough for the reviewer, but for many miRNA studies, this is very often the proof of involvement of a given miRNA. As for the synthetic interactions, well that is something we could eventually address, if need be, but it is arguably the caveat that almost nobody can refute. With all the data we have presented, the most parsimonious interpretation of these data is that the loss of *mir-1* leads to a derepression of *tbc-7*, which in turn causes a loss of post-dauer fertility, in the context of the dauer stage.

* In the experiments in Figure 6 where the authors express versions of *tbc-7* with and without binding site mutations, the experiment is not easy to understand. In the text, it sounds like the authors made a mutation of the endogenous binding site, but in the figure, it looks like they are doing overexpression with transgenes. Given that the authors are looking at the function of a type of repressor and a possible target that are dose dependent, these experiments should be explained and controlled much more carefully. I would expect that even in the wt 3'UTR version, *tbc-7* is overexpressed from a multi-copy transgene. An allele with the *mir-1* binding site mutated was already generated by Gutierrez Perez et al. Sci Adv 2021, the authors should use that allele to assess whether *mir-1* repression of *tbc-7* is meaningful in this context.

In our manuscript, we used the expression of multicopy transgenes to study the effects of *mir-1* and *mir-44* regulation on *tbc-7* expression levels. As a control, we show that the overexpression of wild-type copies of *tbc-7* in the neurons of *daf-2* control animals do not negatively affect the post-dauer fertility (Fig 4D). The only situation when the *daf-2* control animals are post-dauer sterile occurs when the *mir-1* and *mir-44* regulatory region of *tbc-7* 3'UTR is deleted in the transgene. Thus, we argue that the sterility phenotype is due to a loss of *tbc-7* regulation by these two microRNAs rather than it being a confounding phenotype from the overexpression of the transgene itself.

The *tbc-7^{NotI}* mutant from the abovementioned Gutierrez Perez et al. Sci Adv 2021 publication is already very similar to our 3’UTR deletion mutant. In both mutant animals, the *mir-1* seed sequence is disrupted, presumably preventing *mir-1* from binding and acting on the *tbc-7* mRNA. It is unclear what this new experiment would add beyond

what we have already shown, and on top of it, this new deletion variant might also be subject to the same caveats that the reviewer has voiced.

* It is also unclear why the authors use a ubiquitous driver for the experiments in 6D and E, and not a neuronal one.

As suggested by the reviewer, all experiments using the ubiquitous promoter *sur-5p* were repeated with a neuron-specific promoter *rgef-1p* in Figure 4D and 4E.

* Same problem in Figure 7 E-F as before with the experiment from figure 6. It is not easy to understand exactly what the authors did.

In figure 5D, we used the wild-type TBC-7 as a control in *daf-2* control animals. The overexpression of TBC-7 in control animals did not have a negative effect on the post-dauer fertility (in Fig 5D). Only when a non-phosphorylable variant of TBC-7 was expressed in *daf-2* control animals did we observe a loss of post-dauer fertility, suggesting that it was the misregulation of *rab-7* activity by *tbc-7* that caused this defect.

The same situation seems to be occurring in Figure 5E. When we express wild-type TBC-7 in the *aak(0)* mutant and it does not affect the post-dauer fertility as compared to the *aak(0)* control (Fig 5E). However, when we express a phosphomimetic variant of TBC-7 in the *aak(0)* mutants, the post-dauer fertility was restored to a high level. We presume that the catalytically dead TBC-7 protein is binding active RAB-7. However, since it is unable to hydrolyze the GTP, it instead acts as a protector of GTP-bound RAB-7 by preventing a catalytically viable TBC-7 protein from inactivating RAB-7.

As suggested by the reviewer, all experiments using the ubiquitous promoter *sur-5p* were repeated with a neuron-specific promoter *rgef-1p* in Figure 5D and 5E.

Minor concerns
* Most bar graphs lack error bars.

In this study, we use Marascuilo's procedure as a statistical test for % egg laying animals/post-dauer fertility. This test can examine the proportions of several populations simultaneously. In Marascuilo's procedure, error bars are not necessary as we are observing frequencies of a discrete phenotype in a large population. That said, since we are examining for a binary phenotype (sterile or fertile), it does not make statistical sense to include error bars as animals are either sterile or fertile. Each 'bar' in the bar graphs were generated from the percentage of a population of animals capable of reproducing. All experiments were repeated at least twice, each time with 50 animals from three independently generated mutant lines to generate a phenotype percentage for each population.

* The authors motivate their looking at *tbc-7* function in neurons by saying that it's a conserved RabGAP necessary for vesicle dynamics in Drosophila (beginning of page

15). However, *tbc-7* is expressed broadly (also intestine and muscles, Nehammer et al eLife 2019) and functions more generally in the endocytic pathway.

In the publication by Nehammer et al eLife 2019, the authors used a 2,026 bp region of DNA upstream of the transcription start site. Given that the entire sequence of *tbc-7* including upstream and downstream sequences is about 16,500 bp and that *tbc-7* has multiple long introns, we believe that using a small sequence of the promoter could eliminate important regulatory regions (eg. enhancers) of this gene that fine tune the expression of this gene. In addition, in the publication by Nehammer et al eLife 2019, we can see GFP expression of the transcriptional reporter in the head and tail ganglia in the larval animal (Fig S2). Thus, we chose to use the fosmid engineering technology instead as it captures most, if not all, of the cis-regulatory regions of *tbc-7* more accurately. A detailed explanation can be found in the text on lines 274-282.

In addition, the ability of the endocytic pathway to generate vesicles from recycling vesicles (RAB-11 activity) and multivesicular bodies (RAB-7 and RAB-35) is well documented.

* There is no experiment showing direct interaction between *tbc-7* and the RABs. This interaction is only assumed but observed results are also possibly explained by other synthetic defects.

As suggested by the reviewer, we attempted to co-immunoprecipitate TBC-7 with RAB-7. However, we were unable to perform this experiment successfully in *C. elegans*, most likely due to the transient nature between RabGAP and Rab-GTPases and the high rate of reaction for GTP hydrolysis. We also attempted to this experiment with a RAB-7 GTP-locked variant with wild type TBC-7, as modeled after yeast two-hybrid screens, but to no success. We also wanted to try this experiment with a catalytically dead TBC-7 variant however the catalytic arginine required for GTP hydrolysis has not yet been identified and is still unknown.

TBC-7 (tbc1d15) acting as a RabGAP for RAB-7 has been reported in mammalian systems (Peralta et al 2010 J Biol Chem) (Zhang et al 2005 Biochem. Biophys. Res. Commun). Thus, our findings that *tbc-7* regulates *rab-7* activity is supported by literature.

Reviewer 2

This paper is a second chapter in the interesting neurohormornal exploration of daf gene control of germline mitosis control in dauers. The neuronal function of *tbc-7* in dauer germline behavior is very convincing. I did like the use of the neuron specific RNAi to study the Rab proteins regulated by *tbc-7*. That was shrewd and convincing. Figure 5, which establishes that *tbc-7* negatively regulates rab-5 in neurons to in turn affect germline proliferation is the meat of the paper and meshes nicely with the discovery of *tbc-7* in figure and table 1.

But perhaps one can view these as partial dauers……that the signal to "look like a dauer" in the hypodermis and perhaps the pharynx is normal but that other dauer signals, such as suspension of the germline program is not sent from the nervous system. ie *tbc-7* functions in the pathway to some neurohormonal signals but not all of them.

Dauer development is a program….without ampk signaling that program is only partial and therefore defective…..i dont think it is useful to think about dauer as stress or not stress…..dauer is a specific diapause to nematodes, but diapauses are widely distributed across the invertebrates…..often a program to adapt to seasonality of food or environment. diapause changes with latitude, with rain drought cycles. It is used by parasitic nematodes for infection……used by many nematodes for dispersal…… sometimes sophisticated, ….for example to hitch a ride on a fruit fly to a rotting fruit…..the fruit fly actually nicks the skin of the fruit to allow the nematode in……the nematode brings a proper microbiome to the fruit to rot it. to make wine too. so dauer is a program…..and not a minor program. Part of living on a planet with seasons, with plants that bear fruit in seasons, etc. Because it is a program, I think the authors would be wise to survey the dauer characteristics of *tbc-7*(*rr166*). is the pharynx closed off? are the dauers SDS resistant?…..These are easy assays. Is the cuticle dauer like….alae? The right collagens? How does *tbc-7* interact with daf-7 or daf-1 dauers? Does it interact with daf-16 or daf-18, the strongest *daf-2* dauer suppressor mutations?

As suggested by the reviewer, all the mutants isolated from the EMS screen, *daf-2* control, and *aak(0)* mutants were subjected to 1% SDS to test their dauer characteristics. All animals used in this study were resist to SDS treatment when allowed to enter the dauer stage (as indicated in the Methods and Materials section in lines 694-696.

Since all the mutants in this study are resistant to SDS we presume that they are, at least morphologically, dauer larvae, we do not believe that a discussion regarding the interactions between *tbc-7* and the daf genes would improve the clarity or the interest of this manuscript.

Weaker parts of the paper:

a. The viewing of histone methylation in the germline is peripheral to this analysis…it is part of the dividing vs non dividing germline phenotype.....I would move the histone modification figure to Supplemental.

As suggested by the reviewer, the figures containing the histone modifications were moved to the supplemental section (Fig S2 and S3).

b. The small RNAi component of the paper is pretty weak and peripheral to the strong points on *tbc-7* and rab-5. It should be trumpeted a lot less and relegated to supplemental figures and just a few sentences in the main results or discussion. The small RNA factors that emerged from the previous PloS biology paper were not miRNA

factors, but instead Argonautes that mediate silencing of integrated viruses in the *C. elegans* genome. So the exploration of *mir-1* comes out of nowhere. And mRNA target prediction programs for miRNAs are very inaccurate——these promiscuous computer programs have essentially killed the miRNA field. Using Target Scan to find that *tbc-7* is a target of *mir-1* is also a problem: target scan uses an imperfect base pairing of miRNAs to target mRNAs to predict binding sites. It uses about 15 base pairs, which is simply not enough bits to really nail mRNA targets. So with Target Scan plus no evidence that the miRNA Argonautes alg-1 or alg-2 are involved, the exploration of *mir-1* is a pretty shaky edifice. So the decision to study *mir-1* is not driven by the previous very interesting discovery that the small RNA binding Argonaute proteins ERGO-1 and HRDE-1 suppress the runaway germline mitoses or sterility of *daf-2*; *aak(0)* mutants. That discovery motivates a search of 26G siRNAs, not miRNAs. The only hint of miRNA involvement was that dcr-1(RNAi) could suppress as well. But Dcr-1 affects both miRNA and siRNAs so the mostly likely explanation is that siRNAs that target endogenous viruses are missing in the ergo-1 and hrde-1 and dcr-1 RNAi strains, which in turn causes transposition in the germline to in turn suppress runaway germline proliferation. This is target tissue escape from some neurohormonal signal that the authors identify *tbc-7* and rab-5 to regulate from neurons. So the miRNA exploration is tenuous and pretty ad hoc.

However, the one target prediction method that I think is powerful is the ligation of miRNAs to their target mRNAs (from the Pasquinelli and Rajewsky labs). I have the excels of those miRNA target mRNA ligations on my computer drive and a search of *tbc-7* did find that *mir-1* was ligated in a Pasquinelli spreadsheet of 2,000 ligations (one needed to search that spreadsheet with C31H2.1 to find the mRNA). In the Rajewsky excel of 3400 miRNA mRNA ligations, *tbc-7* mRNA is ligated to lin-4 and a few other miRNAs but not *mir-1*. I vaguely remember that lin-4 is dauer defective, probably because of the heterochronic reiteration of L1 fates. So the exploration of *mir-1* is reasonable, even if the other genetic support to explore it is suspect. But the *mir-1* phenotypes are very minor and should be moved to supplemental figures.

In Kadekar et al PLOS Biology, 2019, our group showed that the production of endogenous siRNAs was maladaptive for the *daf-2* larvae and therefore when the production of these RNAs were compromised through the knockdown of *ergo-1* and *dcr-1*, there was a suppression of post-dauer sterility. However, this does not imply that microRNAs are not essential in the dauer stage. The requirement for microRNA production in order to enter the dauer stage has been well documented, thus an exploration into the requirement of two microRNAs to maintain reproductive capacity throughout this stage is justified. As for the role of the other small RNA regulators, we have been teasing this apart over the last 3 years and the results are very exciting, but entirely unrelated to *tbc-7* or *rab-7*. The role of these other small RNA pathways is indirect and likely impinges on DCR-1 allocation.

In this study, we used TargetScanWorm as a starting point for studying the regulation of *tbc-7* expression by microRNAs. We verified this prediction with genetic experiments involving the deletion of these predicted microRNA seed sequences (in Fig 4D and 4E).

In addition to our study, the regulation of *tbc-7* by *mir-1* is also reported by Nehammer et al eLife 2019 and Gutierrez Perez et al Sci Adv 2021. In this study, we want to highlight the regulation of *tbc-7* by two microRNAs that act upstream of TBC-7 function, in addition to AMPK phosphorylation that acts directly on the protein. We would therefore prefer to maintain the results obtained with both *mir-1* and *mir-44* and their associated phenotypes as a figure in the main body of the manuscript.

c. The tissue specific RNAi experiments should be presented more carefully: you have expressed rde-1 in neurons for example, but the siRNAs produced by rde-1 in neurons could spread to other tissues that might not have rde-1 but still respond to *tbc-7* siRNAs. So the experiment should be viewed as RNAi not working as well as normal everywhere except neurons, but not that the rest of the animal is RNAi deficient. This is a weakness of all tissue specific RNAi experiments.

Thus the paper should be about *tbc-7* and rab-5 in neurons. A four figure PLoS Biology paper would be appropriate.

Yes, we acknowledge the inherent weaknesses of tissue-specific RNAi studies. Therefore, we performed phenotypical and biochemical analyses on our tissue-specific strains as described in response to the fourth comment by reviewer 1 on the bottom of page 3 and top of page 4. These changes are reflected in S2 Table, S4 Table, and in the text lines 744-758 for the phenotypic analysis; and in S7 Fig and in the text lines 758-761 for the biochemical analysis.

Minor comments

Whatever happened to the *rr267* allele? is failed to complement *rr166*…..should be in the same gene.

rr166 failed to complement the *rr267* allele, suggesting that these two alleles are in the same complementation group and are both alleles of *tbc-7* (as indicated in Table 1). In this study, we chose to analyze the *rr166* as it was the most effective in suppressing the AMPK germline defects.

On page 4 of the paper introduction
Fare well……not fair well

This error has been corrected.

page 5
stresses associated with dauer development

This error has been corrected.

Table 1

The right column % wild type like has zero information content…..what is wild type like? the germline? the dauer look? fertility? useless descriptor.

Wild type-like indicates the lack of any somatic phenotypes associated with the loss of AMPK signaling. Such phenotypes which include burst, protruding, or multi vulva have been reported in Kadekar et al PLOS Biology 2019. This modification to the legend description of the table has not been added but it will be should the manuscript be accepted for review.

But the suppression of no fertility is very impressive——these are great mutants. An excellent screen.

It seems that in all of your dauer assays there is a *daf-2* allele. But the figures and tables do not specify. Better to use complete genotypes.

All of your figures leave off key genotype information that makes it hard to understand the experiment without reading the legend. It is better to have figures that have more information for decoding the different columns all in the figure.

In response to the reviewer's suggestion, the full genotypes of all the controls are added in every figure legend. Adding the full genotype of every strain in the graph would clutter the figures.
