# Supplementary material for: Germline stem cell integrity and quiescence are controlled by an AMPK-dependent neuronal trafficking pathway
Source: PLoS Genet. 2023 Apr 14;19(4):e1010716. doi: 10.1371/journal.pgen.1010716 (PMC10132661; doi:10.1371/journal.pgen.1010716)
Supplement: Attachment — Submitted filename: PLOS Genetics Response to Reviewers Comments.docx [file pgen.1010716.s017.docx]

**PLoS Genetics Response to the Reviewers**

**Comments to the Authors:**

Reviewer #1

In the manuscript entitled “Germline stem cell integrity and quiescence are controlled by an AMPK-dependent neuronal trafficking pathway”, Wong et al. present the next chapter in our understanding of how AMPK coordinates dauer diapause programs in C. elegans. During early larval stages, unfavorable conditions (or reduced insulin signaling in the daf-2 mutant) drive animals to enter the dauer diapause stage. During diapause, dauer larvae remodel their bodies to become stress resistant and pause their metabolic and reproductive functions until conditions improve for dauer exit. The Roy lab has shown that AMPK is required for germline quiescence and genome integrity during dauer and is specifically required in neurons and the excretory system to regulate these phenotypes. In the absence of AMPK, dauer animals continue to proliferate their germ cells and they lose their germ cell identity. As a result, postdauer adults are sterile. In this manuscript, the authors present evidence the AMPK regulates the RabGAP TBC-7, whose activity regulates the Rab GTPase RAB-7. Inactive RAB-7 is associated with germline quiescence in dauers and postdauer fertility. They show evidence that AMPK phosphorylates TBC-7 to regulate its activity. In addition, the authors show that miR-1 and miR-44 contribute to dauer germline quiescence by playing a role in the downregulation of tbc-7 transcripts. Most interestingly, rescue experiments and tissue-specific RNAi indicate that AMPK, TBC-7, and miR-1/-44 are acting in neurons to regulate the germline during dauer. Future work dissecting the AMPK-dependent signals from the neurons that regulate dauer germline quiescence will be interesting. This manuscript is a significant step in understanding the mechanism of how AMPK regulates dauer phenotypes.

This version of the manuscript has made numerous revisions in response to the previous review. I think that the data presented is compelling, and I do not suggest any additional experiments. However, I do have some suggestions about data interpretation and conclusions which are listed below.

1) The results of the reverse genetic screen in this manuscript are intriguing. To varying degrees, the mutant alleles rescue the postdauer sterility of aak(0) mutants, but they continue to proliferate their germ cells during dauer. Complementation group 3 is particularly interesting in this respect. Since the chromatin marks are largely rescued by the rr166 allele, these data suggest that loss of germ cell identity, and not continued proliferation of germ cells in dauer, is what causes postdauer adult sterility. The rr289 is a great example of this, where the number of germ cells is the same as aak(0) mutants, but 61% of the postdauer adults are fertile. The continued germ cell proliferation may also be contributing to the lack of rescue of lipid storage phenotypes by these alleles, since the stored lipids are likely mobilizing to the germline to support these newly proliferated germ cells. The authors should consider these nuances in interpreting their data, since it points to another AMPK regulated pathway regulating the proliferation of germ cells in dauer.

This interpretation of a loss of germ cell identity rather than the continued proliferation of the germ cells during the dauer stage as a cause for the post-dauer sterility has been included in the discussion section in lines 645-6658. This paragraph includes a discussion about the allele *rr289*, which also causes germline hyperplasia during the dauer stage, but is still post-dauer fertile, suggesting that it may be a separation of function mutation. We also included a discussion of why the alleles *rr166* and *rr267* are unable to suppress the loss of dauer survival but effectively block the germline phenotypes of *daf-2; aak(0)* mutants. We contend that the loss of dauer survival is likely due to the misregulation of lipid storage, which, as the reviewer has suggested, could potentially be associated the available energy to drive the supernumerary germ cell divisions. However, data from our previous work is not consistent with this scenario. The hyperproliferation of the germ cells takes place prior to dauer entry, while the lipid hydrolysis defect only becomes apparent much later in the dauer stage. Furthermore, RNAi of key cell cycle regulators (*cdk1*, *glp-1,* etc...) can block germ cell divisions in AMPK mutant dauer larvae with no effect on dauer survival, while expression of *aak-2* in the neurons had no effect on survival, but does rescue the germline phenotypes. All of these observations together suggest that the two AMPK-dependent processes are unlinked, other than the fact that their regulators are controlled by AMPK-mediated phosphorylation.

2) It is not clear to me whether the eggs laid by the aak(0);daf-2;rr166 animals are viable and hatching. Are these animals producing offspring or dead embryos?

All eight of the EMS-generated alleles that we isolated were able to lay viable embryos which developed into fully fertile adults. This clarification has been added to the manuscript in lines 186-187 in the legend of Table 1 and also in lines 179-180 in the text.

3) The tissue specific rescue experiments are done with pan-neuronal promoters, but it would be very interesting to determine which neurons(s) TBC-7 function is required in this context.

We agree with the reviewer that this is an interesting and very important question, and one that we intend to address in the future. However, we believe that this avenue of investigation is outside the scope of the current manuscript, which focuses on the suppressors that we identified using genetic analysis and how the function of at least one of these suppressors is important in the neurons. This in itself is an important finding that we would very much like to share with other investigators, largely due to the curious nature of cell non-autonomy that exists between these two tissues.

4) In the screen to test for Rabs that are acting downstream of TBC-7, it is not clear why the authors only focused on rab-7 when the rab-10 phenotype is almost identical.

Initially, we did not know whether RAB-7 or RAB-10 was the Rab target of TBC-7. So we employed our tissue-specific RNAi system in the *daf-2; aak(0); tbc-7* genetic background to determine if these two Rab proteins were required in the neurons. Our tissue-specific RNAi experiments showed that the compromise of *rab-10* in the germ line reverted the suppression of AMPK germline defects, while its compromise in the neurons had no effect on the post-dauer fertility, suggesting that *rab-10* is only required in the germ line. Therefore, it is unlikely that RAB-10 would be regulated by the TBC-7 RabGAP that we demonstrated to function in the neurons. These data have been included in a supporting figure in Fig S6 (Fig S6 legend in lines 1257-1262) and in the main manuscript in lines 393-401.

5) I agree with the previous reviewer that the rationale for investigating miRNAs in this pathway is not well-justified in the manuscript. MiRNAs and endogenous siRNA pathways are just not the same. I think the manuscript should be changed to reflect the authors’ response to reviewers, such that miRNAs are investigated because “the regulation of tbc-7 by mir-1 is also reported by Nehammer et al eLife 2019 and Gutierrez Perez et al Sci Adv 2021” and that miRNAs are known to regulate dauer phenotypes.

Our initial work on this project did not focus on miRNAs because we had some difficulty dealing with the role of these small RNAs in dauer formation, not to mention that it was difficult to understand how miRNAs might affect chromatin modifications. But genetics pointed us to the miRNA pathway and other groups I have communicated with are also seeing similar effects of miRNAs on chromatin modifications. Nevertheless, we have modified the introduction to the section that introduces the regulation of *tbc-7* by *mir-1* and *mir-44* to better justify this these experiments. In the new introduction, we justify exploring the potential role of microRNAs regulating *tbc-7* by introducing the Nehammer et al, eLife 2019 and Gutierrez Perez et al, Sci Adv 2021 publications. In addition, we also stated that microRNAs are required to enter the dauer stage, citing Alvarez-Saavedra & Horvitz, Curr. Biol. 2010, providing an additional rationale for investigating microRNA function during the dauer stage. These changes have been introduced in the text in lines 454-467.

6) I think the authors should be cautious about concluding that AMPK is somehow regulating miRNA production. They do not show evidence that miRNAs miR-1 and miR-44 levels are changing, and the rescue experiment overexpressing miR-1 and miR-44 in Figure 4E is analogous to an RNAi experiment (and is convincing that these miRNAs regulate tbc-7 transcript levels!). If AMPK were to be regulating the activity of miRNA biogenesis proteins, all miRNAs would be affected and it seems likely you would observe more severe phenotypes. A more conservative explanation would be that miR-1/-44 are acting in a parallel pathway to AMPK to regulate TBC-7 function, thus supporting a model whereby miR-1/-44 are acting upstream to regulate TBC-7, and endogenous siRNA pathways are acting downstream to regulate germline chromatin and gene expression in dauer.

The reviewer has a good point, and we agree that at present, we provide insufficient evidence in this manuscript to conclude that AMPK is somehow regulating the microRNA biogenesis machinery and microRNA production. We removed sections in the manuscript that state that AMPK is in fact regulating microRNA biogenesis and replaced it with more cautious wording that suggests a possible link. These changes are reflected in lines 713-717, 722-731, and 773-780. We also added a more reserved conclusion that AMPK and *mir-1/44* restrict TBC-7 activity/expression in parallel pathways in lines 722-731 as suggested by the reviewer.

Reviewer #2

The authors have addressed my previous comments and those of Reviewer 1 just fine. Ready to publish.

Thank you!

Reviewer #3

This is revised manuscript by Wong et al addressing the discovery of novel suppressors of ampk mutant- induced sterility after dauer, two of which fall in the tbc-7 complementation group. TBC-7 encodes a RabGAP and they expand upon this analysis to identify RABs that contribute to this pathway, and create relevant mutants in rab-7 that are trapped in the active state. They also examine the regulation of TBC-7 and zero in on two miRNAs that appear to regulate tbc-7 based on mutational analysis of the tbc-7 binding sites and overexpression of the miRNAs. They also find that there is a strong AMPK binding site in TBC-7 and create mutants that mimick the phosphorylated and unphosphorylated states, all of which have the expected phenotypes in post-dauer sterility.

Overall the screen is clever and most of the results are substantiated, the manuscript is still very hard to read because of lack of full genotypes in each part of the figure legends in the figures. There are a number of methods that are lacking, making it difficult at times to assess the methodology (when were transgenes vs fosmids used/ what were specific mutations, etc). Further, there are a few cases, where the results are consistent but do not prove a direct interactions, most importantly as to whether AMPK is truly the kinase for TBC-7. Most critical for acceptance is a clarification of these many technical and textual issues. While further experimentation would enhance the paper, softening some of the conclusions might be sufficient.

Specific comments to address prior to publication

• I think it is still confusing to have to read to the end of the figure legend to get the full genotype. I think there would be ways to show that all of the worms have aak or daf-2; aak. Also for the uninitiated, I think it would be helpful in the results to mention that aak(0) = aak-1 aak-2 double mutant.

All the figures in the manuscript and in the supporting documents have been modified to include the full genotypes. We hope that this change will improve the readability of the manuscript for both *C. elegans* geneticists and non-*C. elegans* investigators. In all the figures except for Fig S4, we state that the animals have the *daf-2(e1370)* allele.

An extra sentence in the beginning of the results section was added to clarify that mutants lacking AMPK signalling have a deletion of both catalytic subunits *aak-1* and *aak-2* and are thereafter referred to as *aak(0)* in lines 167-170.

• All methods should be described in the text itself and should not simply be referenced.

In accordance with the reviewer's suggestions, a detailed description of all the methods including all relevant primer sequences and all transgenic animals (genetic mutants or animals carrying transgenes) have been added to the manuscript in the Materials and Methods section in lines 781-1019.

• There is no description of the how the TBC-7::GFP fosmids were constructed, nor any of the transgenes. This information must be included for publication.

A new section in the Materials and Methods have been added to detail the construction of all the relevant transgenes created for this manuscript. Specifically, more details on how the RAB-7 GTP-locked variant, deletion of the *tbc-7* 3’UTR, and the site directed mutagenesis of the predicted AMPK phosphosite on S115 of TBC-7 have been added, including all relevant primer sequences. These changes can be found in lines 923-965.

A detailed description of how the fosmid containing a wild-type copy of *tbc-7* translationally fused to GFP and also the procedure for the microinjection, concentrations of plasmids/fosmid, and genotypes were included in the Materials and Methods section in lines 907-921. Transgenes were injected at a concentration of 15 ng/μL with some exceptions that are listed in lines 793-798.

• I cannot find a description of the amino acid changed in the RAB-7 GTP locked variant. How was this made and what it the evidence that it has this structure?

The mutation Q67L and Q68L creates a GTP-locked variant of RAB-7 in HtTA1 cells and *C. elegans*, respectively. This mutation was first introduced by Méresse et al, J Cell Sci 1995. In Méresse et al, J Cell Sci 1995, they showed that the introduction of this mutation changes the localization of RAB-7 in HtTA1 cells. In Chotard et al, Mol Cel Biol 2010, the authors introduced the same mutation in the *C. elegans* orthologues, RAB-7 Q68L and found that mutants expressing this GTP-locked variant of RAB-7 displayed larger intestinal vesicles. These phenotypes suggest that this point mutation in the protein sequence would generate a catalytically dead variant of RAB-7, rendering it locked in its GTP-bound state. This information was added to the manuscript in lines 435-441.

In our study, we cloned the RAB-7 GTP-locked gene directly from the plasmid used in Chotard et al, Mol Cell Biol 2010 and inserted the GTP-locked RAB-7 variant in between a neuronal *rgef-1* promoter and a wild type *rab-7* 3’UTR. A detailed explanation of the engineering of this plasmid is included in the Materials and Methods section in lines 923-930.

• It is unclear in Figure 2D how ANY fertility in the aak(0) germline RNAi is considered not statistically different from complete sterility. If this were identified in the genetic screen, you would have kept it as a putative suppressor. This simply means that the numbers are too low to show significance. I suggest using a Mann Whitney test for this analysis.

For testing post-dauer fertility, we use the Marascuilo procedure as a statistical test. This test can examine the proportions of several populations simultaneously. In Fig 2E, the germline-specific RNAi of *tbc-7* in the *daf-2; aak(0)* background is compared, not only to the germline-specific RNAi of L4440 in the *daf-2; aak(0)* background, but also to every other column. Thus, if this were a chi-squared test, any post-dauer fertility would be statistically significant compared to the 0% post-dauer fertility of *daf-2; aak(0)* animals. But since the Marascuilo procedure will compare proportions to every other pair and determine which proportions are statistically significant, the low fertility of germline-specific RNAi of *tbc-7* in the *daf-2; aak(0)* background is compared to the high fertility of neuron-specific RNAi of *tbc-7*, therefore the test will determine the low post-dauer fertility of germline-specific RNAi of *tbc-7*.

As suggested by the reviewer, we repeated the analysis with the Mann-Whitney U test and found that 0% and 11% tested significantly different even at the 0.01 significance level. However, we do not believe that the statistics have any biological relevance given the difference in the post-dauer fertility with the same *tbc-7* RNAi treatment between the germline-specific and neuronal-specific RNAi or between germline-specific RNAi and whole animal. If we use the Mann-Whitney U test to compare between germline-specific and neuronal-specific with the *tbc-7* RNAi treatment, it also tests as significantly different at the 0.01 significance level. Therefore, we believe that using Marascuilo procedure as a statistical test is most suited for this type of analysis. Regardless, together with the imaging in Fig 2B and 2C, and the genetic data from Fig 2D, we believe that *tbc-7* is working in the neurons to regulate the germ line during the dauer stage.

• The prior studies showed potential roles for small RNA pathways but technically did not address in which tissues these genes were acting. Rather the previously addressed where AMPK was acting to control germ line chromatin. Thus, I have issue with the statement that “Our previous findings hinted that small RNA homeostasis is misregulated in the germ line during the dauer stage in AMPK mutants,” since there is no evidence either way that the activity was in the germ line. Indeed their attempt to link tbc-7 to a small RNA pathways undermines this statement since they showed earlier in the paper that tbc-7 is required in the neurons!

We agree that there is insufficient evidence to suggest that AMPK is directly regulating small RNAs in the neurons or in the germ line. Therefore, the paragraph in question that suggests that AMPK might directly impinge on small RNA biogenesis to produce either endogenous siRNAs or microRNAs has been removed. To justify the exploration of small RNA regulation of *tbc-7*, we introduced two other publications by Gutierrez et al, 2021 and Nehammer et al, 2019 that demonstrated that the orthologue of *tbc-7* is indeed regulated by *mir-1*. These changes are reflected in the manuscript in lines 454-467.

We also removed any statements that conclude that AMPK directly phosphorylates microRNA biogenesis components or influencing the production of small RNAs during the dauer stage. These changes are reflected in lines 713-717, 722-731, and 773-780. We added this clarification in the figure legend of Figure 6 in lines 773-780.

• Since a prior reviewer mentioned the use of alternative methods to find miRNA targets that also substantiate the examination of miR-1, it seems reasonable to include this as support for their study.

As suggested by a prior reviewer, we looked into alternative methods that were used to identify any association between 3’UTRs and microRNAs. In the dataset published by Broughton J. P. et al, Mol Cell 2016, we noted that a direct interaction between *mir-1* and the 3’UTR of *tbc-7* was indeed identified. These details are described in the text in lines 454-467.

• It would be beneficial to have a more detailed description of the mir-1 mir-44 phenotypes on day 1 and day 7 of dauer since prior studies show that the germ lines do not continually proliferate in dauer. In these mutants do they fail to proliferate early but show delayed divisions?

A more detailed description of the phenotypes of the *mir-1* and *mir-44* mutants on day 1 compared to day 7 was added into the manuscript. After one day in the dauer stage, these mutants display phenotypes similar to *daf-2* control animals. However, after spending a prolonged period of time in the dauer stage, these animals begin to exhibit phenotypes closer to *daf-2; aak(0)* mutants and are post-dauer sterile. We suggest that the extended duration in the dauer stage could result in a loss of germ cell integrity therefore leading to post-dauer sterility. We also clarify that these mutants recover from the dauer stage at a normal pace when compared to *daf-2* control animals and AMPK mutants. These changes are reflected in lines 487-493.

• Also, the authors state: “Our findings suggest that as the animal spends an extended
414 period of time in the dauer stage, the loss of mir-1 and mir-44 allows for tbc-7 transcripts
415 to be actively expressed.” This is actually not directly test in the paper, but can be substantiated with the WT fosmid tbc-7::GFP that they show in Figure 2.

We did not directly test the transcript levels of *tbc-7* in the *mir-1* and *mir-44* mutants. However, we see an exacerbation of the post-dauer sterility by compromising *rab-7* using RNAi in the *mir-1* and *mir-44* mutants, suggesting that these two microRNAs may function in a linear pathway with *tbc-7/rab-7*. We acknowledge that this is a shortcoming of this manuscript and have made it clear that the inappropriate expression of *tbc-7* in the absence of *mir-1* and *mir-44* is an assumption. These changes are found in lines 500-502.

• It would be nice to the images in Figure 5A presented on a single gel to see if there are differences in mobility that might be expected from a phosphorylated protein.

Please see below for a new version of Fig 5A where the TBC-7::GFP in the *daf-2* control animals and *daf-2*; *aak(0)* mutants are presented on a single gel:

We now present our TBC-7::GFP immunoblot on a single panel (from a single transfer membrane). There is only one predicted AMPK phosphosite, which may or may not cause a shift (it is not uncommon that a single phosphate has no effect on the migration of a protein during SDS-PAGE). Its migration does not seem to be affected by the presence of wild-type AMPK activity. Fig 5A containing the individually cropped blots are replaced with bands that are presented as a single gel in Figure 5 on line 578.

•There are some issues with initial descriptions of the tbc-7 alleles:
-The description of the complementation analysis of the tbc-7(0) and tbc-7(rr166) fails to show provide one important details: a phenotype. It appears from the text that they were testing viability alone, but this result would also be attained with a crosses to a wild type gene. In the methods, however, they specifically mention having the worms transit through dauer. Where is the data for these results?

In the complementation analysis between the null allele of *tbc-7*, *tbc-7(tm10677)*, and *tbc-7(rr166)*, we wanted to test if our *tbc-7(rr166)* allele was a null allele. When these two alleles were crossed together, we could isolate viable heterozygous animals, suggesting that our *tbc-7(rr166)* allele is not a null allele, but is instead either, as the reviewer has alluded to, a recessive gf hypermorph, or a hypomorphic allele of *tbc-7*. We agree that a cross between the null allele *tbc-7(tm10677)* with a wild-type copy of the gene would indeed result in viable offspring, but this was not a possibility since all the alleles involved were mutant variants. Since we did not observe any Lon mutants in the F_2_ generation, we are confident that we successfully isolated a heterozygous *tbc-7(rr166)/tbc-7(tm10766)* mutant.

As for isolating our EMS-generated mutants and for all post-dauer fertility testing, we allowed the animals to transit through the dauer stage. However, in the cross to isolate the heterozygous *tbc-7(rr166)/tbc-7(tm10766)* mutant, the aim was not to examine the post-dauer fertility therefore, we did not introduce the *daf-2(e1370)* allele into this cross; we crossed *tbc-7(rr166)* (alone without any other mutations or *daf-2(e1370)*) into the *tbc-7(tm10766)/tmC30* strain to isolate viable animals.

-There is no analysis of whether this is a recessive gain-of-function. The argument in the response to reviewers that they cannot make the aak(0); tbc-7 transhets because they are on the same chromosome fails to note that they aak-2 and tbc-7 are 30cM apart and thus, easy to construct genetically.

We did not conduct any analysis on whether the *rr166* allele is a recessive gain-of-function. Based on discussions with a number of my geneticist colleagues, we could not come up with a reasonable test that would allow us to confidently conclude that this is NOT a recessive gain of function mutation. That said, using Muller's definitions it is almost impossible to eliminate the possibility that either of these alleles, or any hypomorphic allele for that matter, does not act as a recessive gain of function. But, since these types of mutations are quite rare, it seems unlikely that these alleles would fall into this class. Furthermore, since we isolated two independent alleles (*rr166* and *rr267)* that both affect the germ cells in a similar way, that possibility that both of them would be recessive gf alleles would be very unlikely based on the weak selective pressure of this genetic screen and the frequency that recessive gf alleles arise following classical genetic analysis. Taken together, the most parsimonious interpretation is that these alleles act as recessive hypomorphic mutations that result in the compromised function of *tbc-7* in the absence of AMPK signalling.

- Given that rr166 and rr267 are in the same complementation group, it is likely that the mutation in tbc-7 was easier to find because it was likely the only gene with missense mutations in both strains and not the parental. Since EMS causes hundreds of mutations, it is worth commenting that it not so simple as genome sequencing the strain and finding the mutation, as described. In addition, I agree with one of the prior reviewers that at least discussing the nature of rr267 allele here is warranted.

More details on the preparation of the genomic DNA, sequencing process, and the bioinformatics analysis were included in the Materials and Methods. We included details on the alignment of the genomes to the reference *C. elegans* WBcel235 genome and how genetic variants where identified. These changes are found in lines 800-830.

An extended discussion of the *rr267* allele has been added to the manuscript. We included the % of fertile post-dauer animals, post-dauer brood size, No. of germ cells in the dauer larvae, and dauer survival data in Fig 1 on lines 217-229. We also included a discussion on the exact mutation of the *rr267* allele on *tbc-7* and why the *rr166* allele has a more penetrant phenotype when compared to *rr267*. These changes are described in the text in lines 296-303.

-I am little surprised that the authors did not simply recreate the rr166 mutation by CRISPR. This would have eliminated any concerns about background mutations.

From our EMS suppressor screen, we were able to isolate two alleles of *tbc-7* independently. Furthermore, we confirmed that *rr166* is an allele of *tbc-7* through RNAi and the introduction of a wild-type copy of *tbc-7* through a fosmid. We were also able to revert the suppression of AMPK germline defects conferred by *tbc-7* when we introduced a wild-type copy of *tbc-7* in the neurons. We agree that recreating the *rr166* mutation using CRISPR/Cas9 would go one step further to confirm our claims, but because we obtained two alleles of the same gene in our screen the most parsimonious conclusion based on all our analyses would be that *rr166 (*and *rr267)* is/are an allele(s) of the RabGAP gene *tbc-7*.

-A prior reviewer asked for a summary of the post-dauer somatic effects to be included. Considering that the authors mention that rr166 suppresses most of the somatic defects, including a sentence about the phenotypes is warranted, and a reference to the prior paper. This is important given the statements in the following paragraph (line 184-186) about rr166 specificity.

A description of the post-dauer somatic defects have been added in lines 200-203 and lines 191-194. We clarified that AMPK mutants exhibit a diverse range of somatic defects as a post-dauer adult. Many animals die during the recovery period from the dauer stage, while those that survive often show protruding vulva, burst vulva, or multivulval phenotypes.

–In summary of this section about the alleles: the authors should tighten up the complementation analysis and provide information on the nature of the rr267 allele and reiterate the non-germ line phenotypes that are assayed for publication.

Yes, all these changes have been made according to the above comments.

•The use of phosphomimetic and deficient mutants is convincing, but true proof for direct phosphorylation is lacking and would come from either in vitro studies or creation of a phosphor specific antibody.

We acknowledge that this manuscript lacks the biochemistry to show that AMPK is phosphorylating TBC-7 directly during the dauer stage in order to negatively regulate its activity. It has been very difficult to generate recombinant TBC-7 protein for these assays. While we did not directly show through *in vitro* assays or by generating a phosphospecific TBC-7 antibody, our genetic data points to the importance of the Ser115 residue as a key regulatory switch for TBC-7 activity. We included this clarification in the discussion section in lines 745-746.

• It is still unclear why rab-7 and not rab-5, for example, was chosen for further analysis. The authors state that “..when rab-7 was compromised in the aak(0); tbc-7 mutants, the post-dauer fertility was reduced to a level similar to that of post-dauer aak(0) mutants.” However the aak(0) mutant in Fig 1 and S1 how 0% fertility, like rab-5, and not 4% like rab-7 in the aak(0) tbc-7

*rab-5* was not chosen for further analysis because it affected the % of fertile post-dauer animals in *daf-2* control animals. Therefore, when the *daf-2; aak(0); tbc-7* mutants were post-dauer sterile after compromising *rab-5*, it was impossible to conclude whether the sterility was attributed to the loss of its RabGAP target, or that the loss of *rab-5* in any genetic background causes sterility or dead/sick animals. Therefore, any Rab proteins that caused post-dauer sterility in the *daf-2* control animals were eliminated from further analysis.

Furthermore, TBC-7 (tbc1d15) is a RabGAP for RAB-7 in mammalian systems (Peralta et al 2010 J Biol Chem) (Zhang et al 2005 Biochem. Biophys. Res. Commun). Thus, our findings that *tbc-7* regulates *rab-7* activity is supported by data obtained using other cell/developmental models.

• Why was rab-5 omitted from Figure 3B?

*rab-5* was omitted from Fig 3B because it also caused post-dauer sterility in *daf-2* control animals. Any Rab proteins that caused post-dauer sterility in the *daf-2* control animals were omitted from this graph because it would be difficult to conclude whether the sterility was attributed to the loss of a *tbc-7* Rab target or from the loss of that particular Rab gene function.

• The conclusion that “its expression in the neurons must be targeted by AMPK during the onset of the dauer stage to instruct. the germ line to execute quiescence” is not supported by the data at this point in the paper, at least. There is as yet no evidence that TBC-7 works downstream or is a target of AMPK, rather the data thus far simply supports that it can suppress loss of AMPK function.

We agree with the reviewer that there is not yet enough evidence at this point to indicate that TBC-7 works downstream or is a target of AMPK. This sentence has been modified to simply state that *tbc-7* functions in the neurons to regulate the germ line during the dauer stage in lines 353-357.

Minor technical details that should addressed
Line 282, you should mention how big the upstream and downstream regulatory regions are in the fosmid.

The sizes of the upstream and downstream sequences that could potentially contain regulatory elements have been included in the text in lines 323-326 and also in the Materials and Methods in lines 910-915.

Line 85: Paradis and Ruvkun does not address oocyte development and fertility

The reference has been changed to Tissenbaum and Ruvkun, Genetics 1998. This publication explores the loss of fertility in *daf-2* and *age-1* mutants when switched to high temperatures most likely due to the loss of insulin-like signaling. This change is reflected in lines 99-103 and in the references in lines 1170-1172.

Line 323-325: I think it is worth saying that the activity of TBC-7 is inferred since technically you have not measured the activity.

This sentence has been modified to state that the activity of TBC-7 as a predicted RabGAP is assumed or inferred. This change is reflected in lines 373-375.

daf-2; aak(0); tbc-7 since aak(0) is on the X as is tbc-7, the genotype should be written daf-2; aak(0) tbc-7 or daf-2; aak(0), tbc-7 (though this is often confusing and not preferred)

*aak(0)* is composed of two genes - the *aak-1(tm1944) III* on chromosome III and *aak-2(ok523) X* on chromosome X. We use *aak(0)* to indicate a complete lack of AMPK signalling. Since *aak(0)* is mutant for two genes on two different alleles, we keep it on a ‘separate’ chromosome when writing the genotype. We have also indicated this in the Materials and Methods section on lines 787-791.

In figure 3B, the control genotype should be listed in the key. It was unclear, for example that this was the data to support the statement, “Indeed, rab-7 RNAi during the dauer stage caused post-dauer sterility.” (lines 335-336)? Both the figure was not reference and it was unclear the figure was the rab-7(RNAi) without daf-2;aak(0);tbc-7.

The genotype of the control animal, *daf-2*, has been added to Fig 3B in the key to better indicate that the white bars are *daf-2* animals, while the grey bars are *daf-2; aak(0); tbc-7* mutants. A “RNAi” label has been added to the x-axis of Fig 3B to clarify that these genes are being compromised using RNAi and are not genetic mutants containing these deletions.

Additional figure references have been added in this section in line 386-387 to justify the claim that *rab-7* RNAi causes post-dauer sterility during the dauer stage in *daf-2; aak(0); tbc-7* mutants.

Mutagenesis Methods:
What does F1 animals were isolated to …. does that mean that they were individually plated?
Am I reading correctly that the F2 worms were only at 25°C for 48 hours. So they never actually molted into dauer, but went into L2d and then were recovered

The F_1_ animals were not individually plated. They were isolated to separate the F_1_ generation from the P_0_ generation. The F_1_ generation was allowed to become gravid before they were synchronized to obtain the F_2_ generation that could be homozygous for the EMS-generated mutations. The F_2_ generation was assessed for their ability to suppress any AMPK germline defects. These clarifications have been added to the Materials and Methods in lines 800-830.

The F_2_ worms were allowed to enter the dauer stage (48 hours at the restrictive temperature-25°C) and then spent another 48 hours in the dauer stage, for a total of 96 hours at the restrictive temperature. This clarification has been added to the Materials and Methods section in lines 807-809.

The authors should include a caveat to their Western blot analysis of TBC-7 in AMPK mutants that although the levels do not change, they cannot rule out that protein levels are not changed only in neurons, where both proteins appear to act together to control germline quiescence.

This caveat in our Western blot analysis of TBC-7 levels in *daf-2* control and AMPK mutants has been included in lines 575-577. We stated that there is a possibility that the protein levels of TBC-7 might be affected by the lack of AMPK in tissues other than the neurons.

Textual issues that should be addressed
Line 82: “Both starved C. elegans, or…” no comma. I also do not understand this statement since daf-2 mutants are fertile. I think you are trying to discuss what happened during dauer, but this is totally unclear.

The comma has been removed in this sentence. *daf-2* mutants are not sterile. Tissenbaum & Ruvkun (Genetics, 1998) show that *age-1* and *daf-2* mutants that are exposed to high temperatures during their L4 larval or early adult stages are infertile and do not produce oocytes, presumably due to the lack of insulin like signaling. These clarifying details have been added to this sentence in lines 99-103.

The paragraph in the introduction (Line 114 -126) is confusing. It seems to be setting up a non-germ cell autonomous role for AMPK but then Line 128 is not “surprising” but rather expected since we already know it can act in other tissues. The idea that the sensing can be in neurons could regulate the germ line is reasonable, but the rest of the paragraph is non-specific about which tissues AMPK is in.

We have corrected this oversight. We removed the statement that AMPK function in the neurons is surprising. This change is reflected in line 147.

We also added the specific tissues where we know AMPK is functional. AMPK works in the neurons and the excretory system to suppress the AMPK germline defects. This change is reflected in lines 135-137.

Lines 175-178: These ideas are not intuitively connected: “Moreover, rr166 also suppressed most of the post-dauer somatic defects, suggesting either that this allele is not unique to the germ line, or that it affects the somatic tissues to adjust cell division timing for the duration of the diapause through its effect on the germ cells.” rr66 can have a somatic function independent of its role in dauer.

This sentence has been reworked to better describe where the *rr166* allele might be required. We stated that the *rr166* allele may not be unique to the germ line and can function in the soma during normal development that does not include the dauer stage. This change is reflected in lines 199-207.

Line 257: tbc-7 lethality needs a reference

There is no literature that states that the homozygous deletion of *tbc-7* is nonviable. Currently, there is only one *tbc-7* allele, *tm10766*, that completely deletes the gene. In our hands and according to the National Bioresource Project of Japan, we are unable to isolate a homozygous deletion of *tm10766*, therefore we presume that the homozygous deletion of *tbc-7* is nonviable. We also confirmed that this allele *tm10766* contains a deletion of the entire *tbc-7* gene through the sequencing of the PCR product generated from a set of primers that flank the deletion region.

Other alleles of *tbc-7*, *tm14007* and *tm15233*, that can be found on the National Bioresource Project consortium can be isolated as homozygous animals however, they only contain relatively small deletions of about 100-300 bp (while transcript lengths of *tbc-7* range from about 2200 to 2500 bps) and have not been confirmed to be null alleles of *tbc-7.* *tm14007* removes one exon that is not predicted to be part of the RabGAP domain. The 148 bp deletion does not affect the reading frame of *tbc-7* either, therefore, we assume that the rest of the protein is translated properly. No exact sequence of the deletion is available for the *tm15233* allele, but the flanking sequencing primers seem to indicate that the deletion is somewhere in the 3’UTR of the gene, therefore, it should not affect any of the exons of *tbc-7*. One additional allele of *tbc-7*, *tm1856* contains a relatively larger deletion of 870 bp deletion is also lethal/sterile. With this information, we presume that a homozygous deletion of *tbc-7* is nonviable. We modified the manuscript to state that the lethality of *tbc-7* deletion mutants is presumed, as no mutants that contain a large or complete deletion of *tbc-7* can be maintained as a homozygous animal in lines 304-306.

Line 261 should read “indicated that it is indeed a”

This error has been reworded in lines 306-308.

Line 287 -288 “Because of the claim that TBC-7 might also function in the body wall muscles during the dauer stage”. should be referenced

We added the reference of Nehammer et al., eLife 2019 for this statement in line 339. We also removed the qualifier “during the dauer stage” because Nehammer et al did not perform their study with dauer larvae.

Lines 288, 291, 400 and 683, TBC-7 should be the gene and not the protein.

These errors have been corrected in lines 339, 344, 479, and 908.

Line 297 “the post-dauer sterility and the germline hyperplasia is” should be “are”

This error has been corrected in line 349.

Line 304 “germ cell” should be “germ line”

This sentence has been reworded in lines 353-355.

Line 302 -306. Run on.

This run on sentence has been split into two sentences to improve clarity in lines 353-357.

Supplemental Line 86 should be TBC-7::GFP

This error has been corrected in line 1274.

The term “germ cell integrity” usually references to genomic integrity not proliferative status or developmental stage. I think state, germ cell proliferation or differentiation would be more appropriate here.

Integrity can refer to a number of things and is not exclusive to genomes. In this manuscript and in Kadekar and Roy, 2019, we use germ cell integrity to refer to the immortal nature of the germline and the capacity of each germ cell to give rise to a gamete, an embryo, and eventually an animal. When the integrity of the germ cell is compromised, for example through the misregulation of chromatin marks in the AMPK mutants (Fig S2 and S3), every germ cell loses the capacity to fertilize or become fertilized, despite having gone through one or more additional divisions. We understand the reviewer's point, but nevertheless would a prefer to keep this term as is, to be consistent with previous publications from this lab.

Lines 373-378 are very confusing since it is not always clear when the authors state “misregulated TBD-7” that they are referring to the daf-2; aak(0) background. Further and again in this section, as earlier in the text, they have not yet proven the TBD-7 is misregulated, per se, in this background, rather they infer it from the phenotypes. The text would be best to stick to the genetic terms for these phenotypes.

We modified the sentence to clarify that TBC-7 activity is misregulated only in the absence of AMPK in line 445.

We believe that we have decisively shown that TBC-7 activity is misregulated in the absence of AMPK, as the disturbance of TBC-7 expression through either RNAi or genetic mutations alleviates the AMPK germline defects. Furthermore, we have shown that *rr166* is most likely a hypomorphic allele that can suppress these defects. Altogether, these data are consistent with the possibility that *tbc-7* activity during the dauer stage is maladaptive for the animal and that *tbc-7* must be attenuated in order to preserve reproductive competence throughout the dauer stage.

Line 403-405 would benefit from precise genotypic descriptions

The entire section has been modified to include full genotypes and alleles in lines 478-
502. We also clarified that these mutants either contain a deletion of *mir-1(gk276)* or a deletion of the *mir-44* family (*nDf49* allele).

Line 448: this needs a figure reference

A figure reference of Fig 4D has been added to the end of this sentence in line 535.

Line 451 should state in neurons since the rgef promoter is used for this analysis.

We clarified that it is an enhanced neuronal expression of *mir-1* or *mir-44* in line 538.

Line 500 phosphomimetic spelled incorrectly

This error has been corrected in line 597.

Line 509-510 is a repeat of 468-470.

The sentence restating the bioinformatic analysis to identify the Ser115 phosphorylation site has been removed from lines 509-510. This information was integrated into the next sentence in lines 610-611s.

Line 554: fix “but not it is not”

This error has been fixed in line 669.

Intertissular does not appeat to be a word and should be replaced with “intertissue”

This error has been corrected in line 765.

Line 705 “were transited” should be “were made to transit”

This error has been corrected in line 839.

Line 707 should read “to determine if…”

This error has been corrected in line 847.

Reviewer #4

Starting from an unbiased genetic screen for suppressors of certain phenotypes associated with loss of AMPK, the manuscript details a role for the RabGAP tbc-7 in neurons. The supposition that tbc-7 is required in neurons is based on heterologous gene expression and tissue-modulated RNAi, as well as testing and ruling out other tissue over-expression (muscle, excretory cell) that were previously identified as sources of non-autonomous AMPK effects. An RNAi survey of RABs identifies a particularly relevant target, RAB-7, that phenocopies loss of AMPK when mutated and partially suppresses loss of AMPK when locked in the GTP-bound active state. Further, TBC-7 bears potential AMPK substrate sequences that appear functional based on substitution with non-phosphorylatable or phosphomimetic amino acids. Based on sequence analysis of the tbc-7, 3’UTR sequence, a role for mir-1 and mir-44 is also explored, revealing regulation important after a long dauer duration.

The results are novel and of general interest to many, including those interested in diapause, AMPK function, germline development, and cellular quiescence. In general, the conclusions are supported by the experiments (however, see below). Further, the work and the thoughtful discussion open exciting questions about how RABs regulate signaling from neurons to the germ line.


Comments and suggestions:

1. Screen, Table 1 and identification of tbc-7:
a. Table 1 – Are these averages? What is N? Explain “wild type-like” designation. Why does “no. of germ cells” for aak(0) differ between Table 1 and Figure 1? A table legend would be helpful.

Yes, the data from % egg laying post-dauer animals, no. of germ cells in dauer larvae, and % wild type-like are presented as an average from the quantification of three independent trials, each with 50 animals. This clarification has been added to all the appropriate figure legends.

Wild type-like indicates the lack of any post-dauer somatic phenotypes associated with the loss of AMPK signaling, such as protruding vulva, burst vulva, multivulva, or premature death during the recovery period from the dauer stage. These changes can be found in lines 191-194 and lines 200-207. A table legend has been added in lines 183-197 immediately following Table 1 in the text.

The difference in the number of germ cells in the *daf-2; aak(0)* mutant between Table 1 and Figure 1 is due to combining data from multiple authors. The data has been corrected now in Table 1 to read 153 germ cells instead of 116.

b. Which specific amino acids are mutated in the two tbc-7 alleles (this information is available from the WGS, so maybe just include it)? Do the specific changes lend any suggestion as to how they affect the GAP function? Does molecular change in rr267 suggest why this allele may be a less efficient suppressor?

The *rr166* allele is a missense mutation from serine 520 to phenylalanine. The *rr267* allele is a missense mutation from phenylalanine 156 to leucine. The S520F mutation is in the TLDc domain. While the F156L mutation is in an exon, it does not correspond to any predicted protein domains. This difference in location could account for the difference in the penetrance of the alleles in suppressing the AMPK germline defects. Perhaps the *rr267* F156L mutation does not affect the overall function of the TBC-7 protein as much as the *rr166* S520F mutation. The text has been modified to include this information in lines 296-303.

c. While the best proof for rr166 being the causal suppressor mutation would be to reintroduce the same mutation into the starting background via CRISPR/Cas9 gene editing, the complementation rescue with the fosmid together with the RNAi phenotype are pretty convincing. Nevertheless, the authors may wish to include in Figure 1 data with both the rr166 and rr267 alleles. In particular, Figure 1D rr166 strain is quite remarkably identical to the aak(0) and would be bolstered by a similar pattern of effects on phenotypes shown in Fig 1 A-C but not D.

Figure 1 has been modified to include data from both the *rr166* and *rr267* alleles. These changes have been implemented in Fig 1 in line 217-229 and in the text in lines 212-215.

2. Figure 1 (and other figures):
a. To aid the non-cognoscenti, I also suggest more descriptive Y axes in Figure 1 and elsewhere in the manuscript. Consider (A) Post-dauer % of egg-laying animals, (B) Dauer germ cell number. Also consider putting full relevant genotypes in the figures and strain names in the figure legends. At least indicate on the figure that all strains also carry daf-2(e1370)? Figures in previous papers from this lab include necessary genotypes. It is very confusing to figure out what is being tested without combing through the figure legends.

In response to the reviewer's comment, we replaced all y-axes labels from % of egg-laying animals to % of egg-laying post-dauer animals and from No. of germ cells to No. of germ cells in dauer larvae. We also added full genotypes into all the figures and figure legends to improve clarity. These changes can be found throughout the text and in the supporting information.

b. Often n is indicated as “n=50”. Are all of these in one trial? Please state for each figure.

Each experiment was repeated at least three times with 50 animals in each trial. All the figure legends in the text and the supporting information have been modified to reflect the protocol more accurately.

3. Fig. 4: Reword the Figure title since the function of mir-1 and mir-44 was not directly tested in neurons, nor was the expression of tbc-7 directly tested.

The figure title for Fig 4 has been reworded to “*mir-1* and *mir-44* are required to maintain germ cell integrity during the dauer stage.” as the functions of *mir-1* and *mir-44* were not directly tested in the neurons nor was the expression of *tbc-7* in the loss of these two microRNAs. This change can be found in line 510-511.

4. Methods: Methods are insufficient and, in addition to specifics below, require editing with attention to italics and other nomenclature.

The Materials and Methods section has been significantly reworked to include many more details on every method and reagent used. The EMS genetic screen and the variant calling from the whole genome sequencing now has many more details. More details have been provided for the complementation analyses. The construction and the concentration of the plasmids have been included. Primer sequences were included where relevant. Extra attention has been given to italics and other nomenclature. These changes can be found in lines 781-1019.

a. Screen – please make screen strategy more accessible to reader. What strain (name + genotype) was mutagenized? Exactly what phenotype was screened for (presumably post-dauer % egg-laying adults?)? Provide more details on WGS analysis.

The strain name and the complete genotype of the mutagenized strain has been added in line 802. The section regarding the generation of the EMS candidates have been completely rewritten to include additional details and to improve clarity in lines 800-816. A new section has been added to provide a detail protocol and on the WGS. We included details on the sequencing protocol and results, as well as how genetic variants from the sequencing were ranked statistically. These changes are in lines 817-830.

b. Complementation – the text requires at least a reference for “complementation analysis was conducted as previously described following injection of myo-2…”. Ideally more details on the complementation analysis would be provided.

A reference for the complementation analysis has been added in lines 833-834. The entire section for complementation analysis has been reworked to include many more details on the complementation analysis, including the setup of the transgenic strains, how the complementation crosses were done, and what parameters were used to assess for complementation. These changes can be found in lines 832-846.

c. Microscopy – indicate how ratio quantification was performed.

The ratios for the fluorescence intensity across the germ line were determined using ImageJ. The ratio was calculated by dividing the immunofluorescence signal of either anti-H3K4me3 or anti-H3K9me3 by the fluorescence signal of DAPI per nucleus. This change is found in lines 976-978s.

d. Site-directed mutagenesis – include all methods on how 3’ UTR deletions and amino acid substitutions were made, including primers, etc.

Details on how the *mir-1* and *mir-44* seed sequences were removed from the *tbc-7* 3’UTR and how the amino acid substitutions on Ser115 on *tbc-7* were performed as well as all the primers used were included in the Materials and Methods in lines 923-965.

e. Transgenes – state exactly what is in the transgenes driven by unc-199, myo-3 and sulp-5 promoters (tbc-7 cDNA?);

These transgenes are driving *tbc-7* cDNA. This change has been reflected in the text in lines 285, 337, 340, 3352, 944, 947, 961 and in the supporting information document in line 68.

f. Strain list – include strains bearing all transgenes (including 3’ UTR, phosphor site mutations, and heterologous expression) in the strain list together worm strain names and plasmid names, and references where necessary.

The strain list has been updated to include all strains used in this study including all transgenes in Table S6 supporting document with table title in line 1338. References were included where necessary.

5. Supplement Tables
a. Table S2 & S4: Legend and/or headings could be improved to help reader understand the set up – table is confusing.

Many more details on the experiment have been added to the heading and table legend for Table S2 and S4. More background on how the tissue-specific RNAi strains were created and how each RNAi phenotype (dumpy, embryonic lethal, or paralysis) was scored is included. These changes can be found in lines 1289-1304 and 1313-1328.

b. Table S3: Also has daf-2 in the background? If so, state.

Yes, all mutants used in this table have the *daf-2(e1370)* allele. This change has been reflected in the Table S3 and in the table legend in lines 1306-1309.

6. Other:
a. Line 412-413, consider restating. That mir-1 and -44 mutants enhance post-dauer sterility of rab-7 RNAi does not necessarily indicate a linear pathway.

We agree with the reviewer’s analysis. We removed any sentences that suggested that *mir-1* and *mir-44* are in a linear pathway with *tbc-7/rab-7* signalling in lines 500-502.

b. Line 543, restate. The screen was perhaps near saturation, but not “indeed saturated” since Groups 4 and 5 are represented by only one recessive allele each.

We agree with the reviewer’s analysis. Instead of stating that the screen was indeed saturated, we suggested that the screen was near saturation. This change can be found in line 641.

c. Line 558. Reference to Kadekar and Roy 2019 – This paper (Fig 8) shows that AMPK expression in neurons and excretory cell can rescue post-dauer egg-laying and germline hyperplasia during dauer. However, I do not see results on premature lethality during dauer (perhaps I missed?).

Kadekar and Roy 2019 does not test whether *aak-2* expression in the neurons can rescue the premature lethality during the dauer stage. Narbonne and Roy, Nature 2009 show that neuronal AMPK expression cannot rescue the premature lethality during the dauer stage. The reference has been corrected in line 658.

d. Lines 707-712. Alert the reader to additional information in S4 Figure.

A figure reference to Fig S5 has been added to the complementation analysis of *tbc-7(rr166)* to *tbc-7(tm10766)* in line 852.
